# Cancer Neuroscience: Innovative Conception and Emerging Strategy of Therapy

**DOI:** 10.1002/mco2.70708

**Published:** 2026-04-01

**Authors:** Ting Wang, Zikai Dong, Yongfei Wang, Ziyi An, Siyuan Wang, Wei‐Lin Jin

**Affiliations:** ^1^ The First Clinical Medical College of Lanzhou University Lanzhou China; ^2^ Institute of Cancer Neuroscience Medical Frontier Innovation Research Center The First Hospital of Lanzhou University The First Clinical Medical College of Lanzhou University Lanzhou China

**Keywords:** brain–body, comorbidity, drug repurposing, neuro‐microbic‐oncology, perineural invasion, tumor microenvironment

## Abstract

Cancer neuroscience has emerged as a paradigm‐shifting discipline that reveals the active role of the nervous system in tumor development and progression. This review synthesizes current understanding of how bidirectional interactions between neurons and cancer cells influence tumorigenesis, metastasis, and therapy response. While earlier frameworks have established the fundamental mechanisms of nerve–tumor interactions, the present study proposes an expanded classification scheme that incorporates two additional mechanisms: perineural invasion as a unique metastatic pathway and neuro‐microbic‐oncology, which incorporates the gut–brain–immune axis into cancer biology. The remodeling of tumor microenvironment is structured around three principal mechanisms: electrochemical signaling, paracrine communication, and neuroimmune modulation. The contribution of these interactions to cancer‐associated comorbidities, including pain, cachexia, and cognitive dysfunction, is highlighted, and their translational relevance is discussed in the context of emerging neurotherapeutic strategies. This review provides an integrated conceptual framework that connects neurobiology, oncology, and immunology, thereby informing the development of nerve‐targeted therapeutic strategies with potential to improve clinical outcomes in cancer.

## Graphical abstract

In this review, we summarize nerve–cancer interactions and conclude two additional interactions, “perineural invasion” and “neuro‐microbic‐oncology.” Emphasizing the important role of nerves in tumor progression. The nervous system also contributes to the development of cancer comorbidities such as cancer depression and cognitive dysfunction. Thus, there are potential applications of targeted nerve–cancer interactions in tumor therapy. This review summarizes the impact of the nervous system on cancer development to assess the status and prospects of targeting nervous system as an anticancer therapy. This figure was created using BioRender (https://biorender.com/)

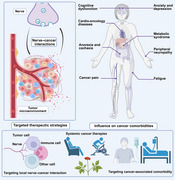

## 1 Introduction

For decades, oncology research has primarily focused on the genetic mutations of tumor cells themselves and the corresponding immune responses. However, over the past two decades, the roles of other cells, organs, and even entire systems in cancer progression have been increasingly recognized. Our understanding of tumors has expanded from simplistic cellular lesions, to interactions between tumor cells and their surrounding cells, and now to viewing cancer as a systemic and multiorgan disease. Given the complexity of these interactions, focusing solely on the role of cells, tissues, or organs may provide an incomplete framework for understanding tumor biology. A broader brain–body communication perspective has therefore emerged as a relevant conceptual approach for studying and managing cancer [1]. As a primary bridge for bidirectional signaling between tumors and distant tissues and organs, the nervous system plays a central role.

Cancer neuroscience has emerged as an interdisciplinary field dedicated to elucidating the complex interactions between the nervous system, tumor cells, the tumor microenvironment (TME), and other organs [2]. This discipline focuses on the local and systemic communication between malignant cells and neural elements and investigates how these interactions regulate cancer initiation, progression, metastasis, and the tumor immune microenvironment (TIM). Over the past two decades, the association between the nervous system and cancer has been increasingly substantiated. Accumulating evidence indicates that neural elements exert complex regulatory functions during tumor development and progression, and tumor innervation is increasingly recognized as a potential emerging hallmark of cancer. However, a framework centered exclusively on the classical hallmarks of cancer does not fully account for the systemic and multiorgan dimensions of tumor biology. This systems‐level perspective provides a conceptual basis for further advances in cancer neuroscience [3, 4].

Although several comprehensive reviews have summarized established modes of nerve–tumor interactions, the rapid development of this field warrants systematic integration of recent advances and a critical expansion of existing frameworks. This review address this need by consolidating knowledge in cancer neuroscience and proposing updated perspectives to elucidate nerve–tumor interaction. Specifically, we incorporate two additional mechanisms, perineural invasion (PNI) and neuro‐microbic‐oncology, into the prevailing framework, to provide a more comprehensive conceptual foundation for future investigation.

This review systematically examines the multifaceted roles of the nervous system in cancer development and evaluates the current status and future potential of targeting neural pathways as anticancer strategies. It summarizes the origins and historical evolution of cancer neuroscience and outlines the physiological basis of the nervous system as a central regulator of systemic homeostasis. The core sections provide an in‐depth analysis of diverse mechanisms of nerve–tumor interaction, including the newly proposed conceptual extensions. The review further examines the distinct neurobiological features of central nervous system (CNS) and peripheral nervous system (PNS) malignancies. It also provides a systematic analysis of cancer‐associated neurological comorbidities. In addition, therapeutic strategies targeting nerve–tumor interactions are comprehensively evaluated, followed by a synthesis of current challenges and future directions in this rapidly evolving field. The overall structure is designed to offer a coherent conceptual framework for understanding cancer neuroscience.

## 2 Cancer Neuroscience: Origin and Development

**FIGURE 1:**
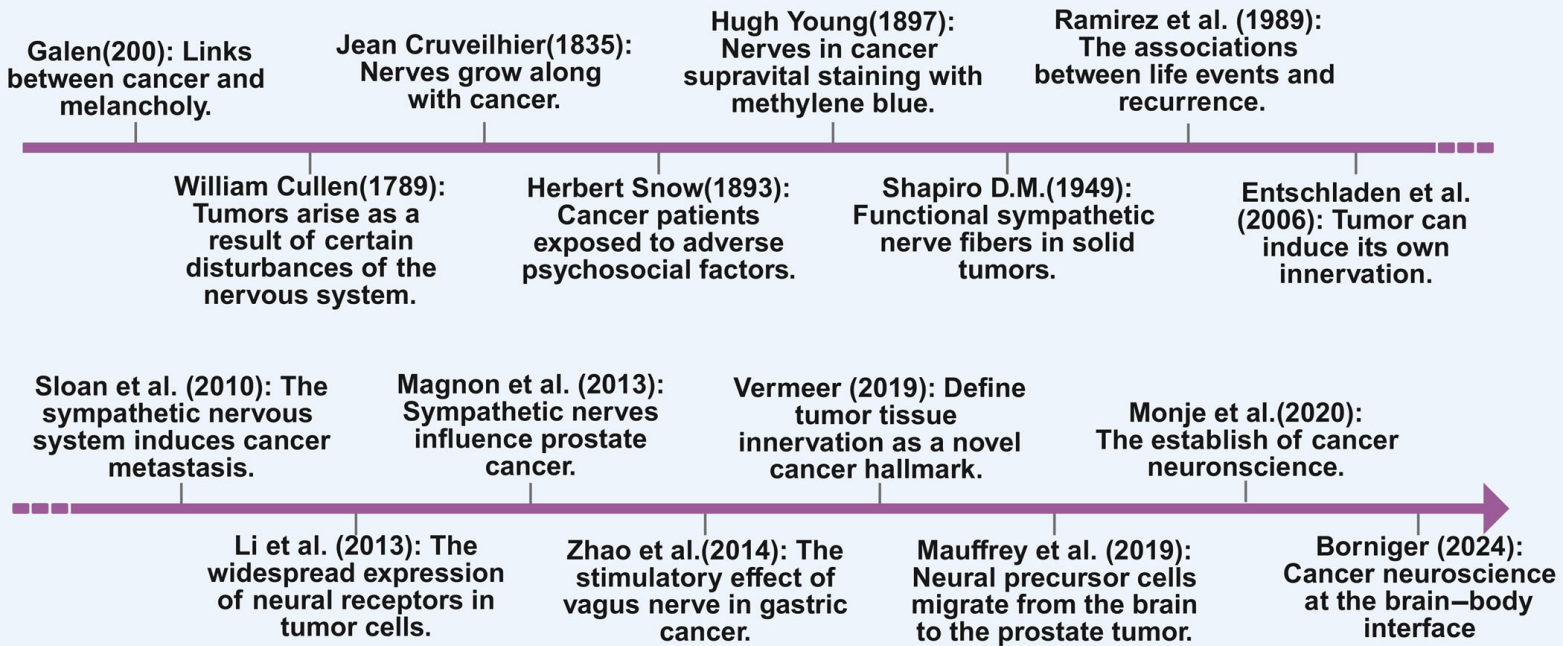
The development of cancer neuroscience. Milestone and representative discoveries of cancer neuroscience. This figure was created using BioRender (https://biorender.com/).

Back to history, the first clue of cancer neuroscience can be retrospect to classical antiquity, when Hippocrates and Galen brought out the hypothesis that there are potential links between cancer and melancholia, referred to as depression in contemporary terminology, resulting from an excess of black bile in the body in the body [5] (Figure 1). For several centuries, this view of cancer as a consequence of melancholy remained prevalent. However, with the emergence of modern anatomy and pathology, the black bile theory was gradually replaced. Although sporadic evidence has suggested associations between adverse psychosocial factors and cancer development, the mechanistic basis of this relationship remains unclear [6]. In 1835, an anatomist, Jean Cruveilhier, first observed a tumor growing along the tortuous course of a nerve, migrating and spreading along the facial nerve. Over subsequent decades, anatomists and pathologists systematically documented the tendency of tumors to grow in close association with nerves throughout the body. Then in the nineteenth century, Paget's soil and seed theory provided the theoretical basis of research in TME [7], supported by discoveries showing that tumor progression is driven by neoangiogenesis and immune cell plasticity [8], while the role of nerves remained largely overlooked. With the subsequent emergence of the concept of the TME, which may be located at the center, periphery, or adjacent to the tumor lesion, it was defined as comprising nonmalignant cells, blood vessels, lymphoid organs or lymph nodes, nerves, extracellular components, and metabolites [9]. The TME concept expanded the scope of oncology from tumor cells alone to the crosstalk between cancer cells and surrounding components, thereby emphasizing the role of the TME in the acquisition of cancer hallmarks [10] (Figure 1).

However, the significance of nerves in cancer was largely ignored for more than 150 years, until Magnon et al. demonstrated in 2013 that, in prostate cancer and surrounding normal tissue, the densities of sympathetic and parasympathetic nerve fibers were associated with poor clinical outcomes in a retrospective blinded analysis, and that nerve fibers contribute to cancer growth and metastasis by sprouting into tumor tissues through axonogenesis [11] (Figure 1). This finding opened a new avenue for cancer research and therapy. The promoting role of the vagus nerve in tumor progression was identified by Zhao et al. in 2014 after a study showed that vagotomy or botulinum toxin type A administration in mice dramatically decreased the incidence and progression of gastric cancer in denervated regions [12]. Subsequent studies confirmed these observations in pancreatic [13], skin [14], and breast [15] cancer, providing strong evidence that tumor tissues are infiltrated by autonomic or sensory nerve fibers that release neuroactive substances capable of binding to receptors expressed on cancer and stromal cells. The possibility of a more intricate neural regulation in cancer is raised by the fact that, in pancreatic cancer, cholinergic nerves and signaling can inhibit tumor progression, whereas adrenergic denervation exerts similar inhibitory effects [16]. Thus, after nearly two millennia, the link between the nervous system and cancer was affirmed, and a novel field named cancer neuroscience has emerged [2]. Tumor tissue innervation is regarded as a unique cancer hallmark because of accumulating evidence indicating that nerves play complex roles in tumor progression [17, 18, 19]. Although the hallmarks of cancer framework defines the characteristics that tumor cells acquire to enable proliferation and dissemination, and has been refined over time, important limitations remain. This theory undervalues the impact of environmental exposures, host pathophysiology, germline genetic diversity, and the interaction between the developing tumor and distant host organs on the development and spread of cancer. According to this viewpoint, understanding cancer's characteristics alone is insufficient to fully explain the various ways that the disease manifests clinically [4]. It is now appropriate to consider cancer as a systemic disease and to embrace its complexity to complement the framework of cancer neuroscience.

In order to gain an updated understanding of how cancer originates and progresses, cancer neuroscience seeks to understand the interactions that occur between the nervous system, cancer cells, the TME, and other organs [20] (Figure 1). This emerging field focuses on the local and systemic interactions between cancer cells and the fundamental components of the nervous system, including neurons, astrocytes, oligodendrocytes, microglia, Schwann cells, and peripheral nerves. Additionally, it examines how these interactions affect tumor development, progression, the TIM, and metastasis [21]. The phenomenon of crosstalk between cancer and the nervous system has been observed to occur in two distinct ways: first, through direct interactions between cancer cells and the nervous system; and second, via neural regulation of other cell types within the TME. The potential for interaction between neural cells and cancer cells may occur within the local microenvironment or through systemic signaling mechanisms, such as the transmission of neurotransmitters within the circulatory system [22] (Figure 1). The significant psychological impact of cancer has already been well established, and the reciprocal interactions between tumors and the nervous system suggest that more integrative research is required. As previously discussed, there may be a connection between psychiatry, psychology, psychosocial biology, and cancer. However, the molecular mechanisms underlying this relationship remain largely unclear. The remaining gaps between oncology, neuroscience, psychology, psychiatry, and the social sciences must be bridged in order to develop a more comprehensive understanding of cancer and, ultimately, to provide the best possible care for cancer patients.

## 3 Nervous System: The Integrator of Systemic Homeostasis

Understanding the role of neural signaling in tumor development requires consideration of the physiological properties of the nervous system as an integrator of systemic functions, including its extensive innervation patterns and multilevel regulatory mechanisms. These features constitute a central basis for the maintenance of homeostasis and provide the theoretical framework for understanding the active contribution of neural pathways to tumorigenesis and cancer progression. The nervous system is broadly organized, similar to the circulatory system, and almost every tissue is densely innervated [2]. It plays a central role in maintaining systemic homeostasis, preserving overall bodily integrity, and regulating responses to both internal and external stimuli. Neuronal activity influences organogenesis, homeostasis, plasticity, and regenerative processes at the systemic level [22]. Because of the widespread distribution of the nervous system and the complexity of its functions, its potential role in tumor initiation and progression can be inferred.

The canonical somatosensory nerves convey stimuli to the CNS for processing, and efferent motor and autonomic nerves transmit signals from the CNS to target tissues, as neural circuitry provides the communication pathway between the brain and the rest of the human body [23]. Since organogenesis depends on appropriate innervation, the importance of nerves in development has become increasingly recognized. It has also been demonstrated that nerves regulate multiple early organogenic processes during embryonic development. In mammals, parasympathetic stimulation occurs concurrently with salivary gland development and maintains epithelial progenitor cells in the salivary gland in an undifferentiated state [24]. The development of the inner ear [25] and airway [26] also require local innervation. Nerves within tissue stem cell niches regulate the proliferation and differentiation of stem cells. The fate of intestinal stem cells is partially determined by the enteric nervous system (ENS), which promotes their differentiation into chemosensory enteroendocrine cells [27]. Signals originating from the sympathetic nervous system inhibit osteoblast function and regulate the recruitment of stem cells to their niches [28]. Peripheral blood vessels, which are under neural regulation, develop, grow, and navigate in parallel with nerves using similar signaling mechanisms [29]. Additionally, innervation contributes to overall tissue regeneration. Surgical denervation of a limb prior to amputation results in healing characterized by fibrotic scar formation and impaired regeneration, with the extent of regeneration dependent on the degree of denervation. Since blastema formation represents the initial response to tissue injury, complete denervation fully inhibits regeneration by preventing blastema formation [30]. Furthermore, nerve deviation at a site of injury can result in the emergence of a supernumerary limb [31].

Nervous system can also communicate with immune system to coordinate the tissue homeostasis. The immune and nervous systems communicate via several mechanisms. These mechanisms include sympathetic innervation of lymphoid organs, the synthesis and function of neuroendocrine peptide hormones, the modulation of immune activity by neuroendocrine signals, the regulation of the hypothalamic–pituitary–adrenal (HPA) axis by cytokines, and both short‐ and long‐range bidirectional communication between the immune and nervous systems [32]. All lymphoid organs receive sympathetic innervation [33]. It is clear that the sympathetic nerves separate from the vasculature when they enter the lymphoid organs. They reach an end near plasma cells and parenchymal T lymphocytes. This interaction gives rise to specialized structures termed neuroeffector junctions [33]. Thus, proinflammatory cytokines released by activated innate immune cells stimulate the afferent vagus nerves, which in turn trigger reflexive activation of the neuronal cell bodies of efferent vagus fibers [34]. The parasympathetic branch of the vagus nerve is responsible for activating the splenic nerves. Acetylcholine‐producing T cells, which suppress inflammation, are recruited upon activation of these splenic nerves. It has been established that innate immune cells express the α1, α2, and β2, subtypes of adrenergic receptors (ARs) [32]. In contrast, adaptive immune cells are the main source of expression for the β2 subtype of ARs [35]. Activation of β2‐ARs suppresses the release of inflammatory cytokines, whereas activation of β1‐ARs enhances their release [36, 37, 38, 39]. Additionally, catecholamines, glucocorticoids, and acetylcholine produced by immune cells can function as local paracrine or autocrine signaling molecules [40, 41, 42, 43].

In summary, the nervous system governs cellular generation, organogenesis, adaptation, plasticity, homeostasis, and tissue regeneration. Because of the analogies between tumor growth and tissue regeneration, the essential role of nerves in cancer is plausible. Early publications have already described the outcomes of denervation in cancer, but these studies were largely overlooked. Similar to development and regeneration, in vivo denervation experiments have been used to initiate research on tumor innervation [12]. Surgical denervation of afferent nerves, or the use of neurotoxic drugs in the tissue, organ, or tumor, has been applied in clinical practice. Although these methods have inherent limitations due to incomplete denervation or nerve regeneration, they still provide strong evidence for the impact of nerves on tumor progression. Similar to regeneration, several trophic factors released by nerves in the TME can promote tumor growth, and conversely, tumor cells release neurotrophic factors that stimulate axonogenesis and nerve infiltration [44]. The tumor–nerve interaction supports the coevolution of tumor and nerve, ultimately establishing a pro‐TME.

## 4 Cancer Neuroscience: Interaction Between Nerves and Cancer

Given the central role of the nervous system in maintaining tissue homeostasis, this intricate regulatory network is frequently co‐opted or dysregulated during tumorigenesis. Tumors are often characterized by enhanced innervation and chronic inflammation, leading to the production of both local and systemic mediators. These mediators can be sensed by both the CNS and PNS [21]. Nerve–tumor interactions are bi‐directional. Neural pathways can regulate tumor progression and cellular behavior, whereas tumors release signals to remodel neural structures and in turn modulate behavioral and physiological responses. Because cancer development is typically a chronic process, sustained tumor‐derived signaling may influence CNS function over extended periods.

### 4.1 The Triggers of Cancer Innervation

**FIGURE 2:**
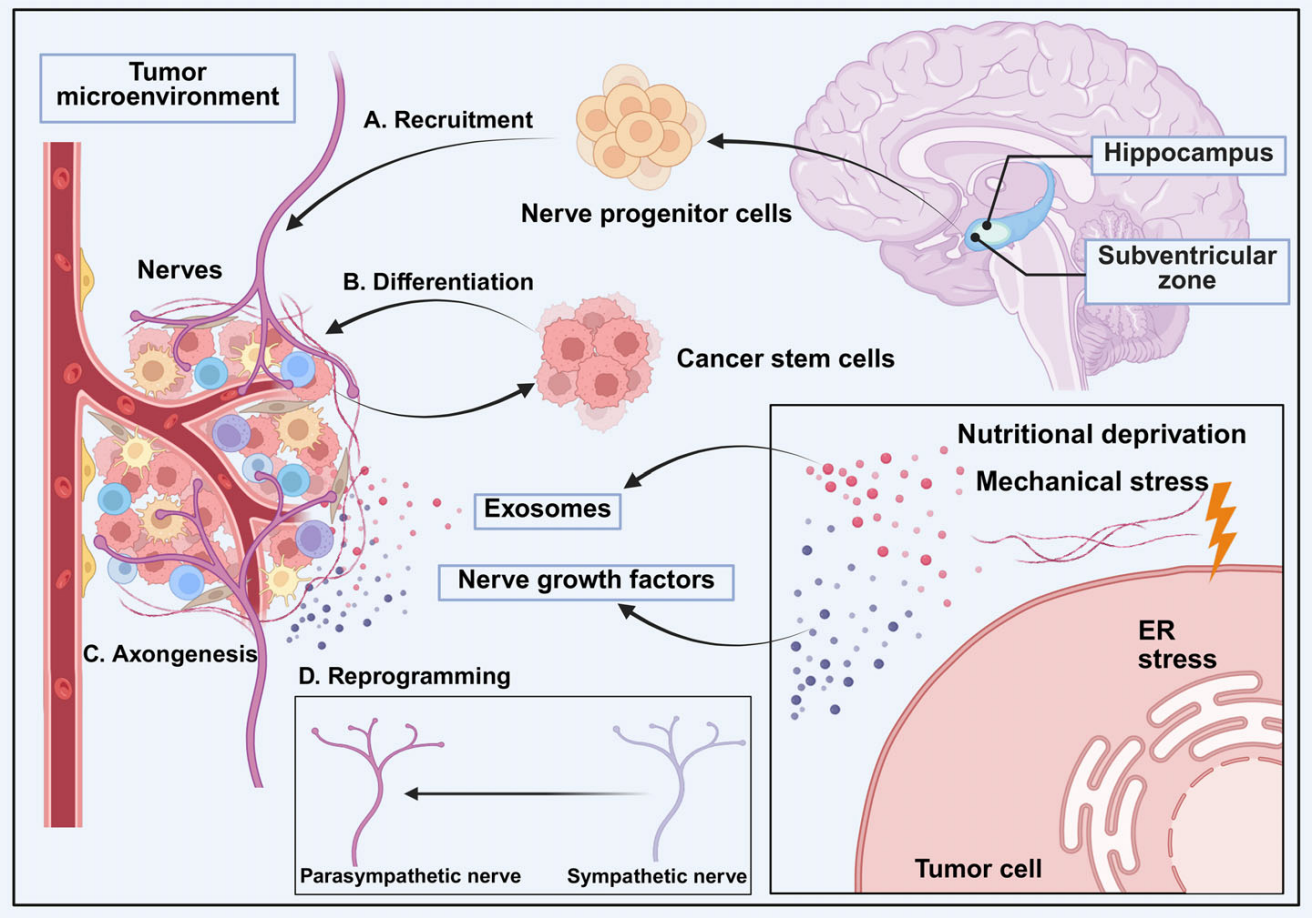
The triggers of tumor innervation. The source of intratumor nerves could be summarized as follows: (A) recruitment of nerve progenitor cells from subventricular zone, (B) differentiation of cancer stem cell, (C) axonogenesis between local nerves and tumors. (D) Tumors could also trigger reprogramming of exist nerves, from sympathetic nerves to parasympathetic nerves, which facilitates tumor survival. And the triggers of tumor innervation could be concluded as: endoplasmic reticulum stress, mechanical stress within the tumor microenvironment, nutrient‐deprivation, and others. This figure was created using BioRender (https://biorender.com/). ER stress, endoplasmic reticulum stress.

Previous hypothesis of the origin of nerves in cancer is that cancer recruits local peripheral neurons that typically innervate the tissue from which they originate (Figure 2). For instance, in head and neck tumors, the crosstalk between trigeminal nervous and oral cavity squamous cell carcinoma cells (OCSCC) with TP53 mutant induce the phenotypic switch of nerves, and adrenergic neo‐neurons that develop from sensory nerves. And surgical ablation of sensory nerves stops the development of these adrenergic neo‐nerves in the OCSCC mice model [45]. Similar to the physiological process, parasympathetic fibers infiltrate tumor tissues whereas sympathetic fibers build up in normal tissues and the tumor margin in prostate cancer. Immunodeficient nude Balb/c (nu/nu) mice were injected with PC‐3 human prostate cells that stably expressed luciferase (PC‐3luc) into their ventral prostate. Histologic examinations show that tumor‐infiltrating sympathetic fibers arise from normal tissue [11]. These evidences show that tumors may use locoregional brain plasticity to support their own development.

However, beside the locoregional nerves, resident nerve subtypes that invade the tumor site are not necessarily identical to those seen in the original tissue (Figure 2). In ovarian cancer, transient receptor potential vanilloid 1 (TRPV1)‐expressing neurons are found to be abundant in tumor area, which do not exist in normal ovarian tissue, indicating that the remote locoregional neurons are recruited in tumor area [46]. In prostate cancer, lineage‐tracing of doublecortin‐positive (DCX+) neural progenitor cells (NPCs) that begin in the subventricular zone (SVZ) shows that SVZ‐derived NPCs have the capacity to penetrate the blood–brain barrier (BBB) and disseminate through the circulatory system, thereby colonizing the prostate cancer [23]. After arriving in the TME, these NPCs undergo maturation, resulting in the formation of TH+ sympathetic nerves, which can promote prostate tumorigenesis [11]. Moreover, the depletion of these DCX+ NPCs has been demonstrated to result in the abrogation of prostate tumor initiation and progression. And it has been shown that the transplantation of DCX+ cells from the SVZ promotes tumor growth and metastasis [47]. In colorectal cancer, NPCs contribute to sympathetic innervation, enhancing metastasis via β‐adrenergic signaling pathways activated by norepinephrine [3, 4, 5]. This concept sheds important light on how cancer could take advantage of typical systemic developmental processes like neurogenesis. It also implies the presence of intricate communications between the CNS and peripheral malignancies. Therefore, the recruitment of these cells to the TME may be due to cancer‐derived substances that are in the bloodstream.

Stem cells maintain the ability of self‐renew and differentiate to offspring of various lineages. When it comes to tumor stem cells, it indicates that one single cell with the capability to form tumor heterogeneity. Furthermore, it is reasonable to believe that the offspring of cancer stem cells may really develop into neural populations inside the tumor that have the capacity to develop into fully functioning nerves in the TME. It is well recognized that cancer stem cells may develop into endothelial cells and pericytes, two components of the TME [48, 49, 50] (Figure 2). The formation of xenograft tumors was inhibited by attenuating the ability of stomach, lung, and colorectal cancer stem cells to produce neurons when implanted intraperitoneally in a model of nude mice [51]. It is imperative to ascertain the necessity of targeting cancer stem cells themselves or the process by which cancer stem cells develop into neurons, to avoid the possibility of cancer becoming resistant to antineurogenesis treatments. In normal conditions, metaplastic tuft cells (mTCs), which are a part of metaplastic ducts, are isolated chemosensory cells that are present all throughout the body. It has been suggested that mTCs represent a population of tumor progenitor cell in pancreatic neoplasia. In pancreatic cancer, mTC transdifferentiate to neural‐like progenitor cells, shifting their role in early‐stage disease from chemosensory‐mediated suppression of tumor growth to an altered functional state. This finding suggests the possibility of other cells in TME transdifferentiating to neural‐like progenitors to promote cancer progression [52].

Under normal physiological conditions, intracellular neuronal signaling changes in response to local biochemical signals—such as growth factors, neuropeptides, neurotransmitters, and morphogens—that can attract axons to target regions govern nerve development. As cancer can hijack the normal developmental processes to promote tumorigenesis, these signals may facilitate the recruitment of cancer. The initial factors of tumor innervation can be endoplasmic reticulum (ER) stress, nutritional deprivation, mechanical stress, and exosomal induction (Figure 2). Given that hypoxia, nutritional deprivation and anticancer medicine can induce ER stress, then tumor cells express and release of brain‐derived neurotrophic factor (BDNF) into the TME, resulting in neurite outgrowth and tumor innervation [53]. And in the nutrient‐poor TME, the lack of amino acid also drives the innervation of tumor. The metabolic crosstalk between axons and cancer cells represents a critical adaptation that facilitates the growth of pancreatic ductal adenocarcinoma (PDAC) in nutrient‐poor environments. Two of the six Ser codons, TCC and TCT, experienced ribosomal stalling as a result of serine deprivation. This allowed PDAC cells to selectively translate and secrete nerve growth factor (NGF) to support tumor innervation. This result was confirmed in mouse model. Mice fed a diet devoid of serine and glycine showed improved innervation and slower growth of exSer‐dependent PDAC [54]. Extracellular matrix (ECM), as the primary noncellular component and a key biomechanical feature of the TME, significantly modulates tumor innervation. These findings indicate that ECM stiffness influences the formation of tumor‐infiltrating nerves in genetically engineered mouse models of pancreatic cancer. The current study examines the integrin–YAP1‐dependent mechanotransduction that underlies the neurotropic effects of ECM rigidity [55].

Although those mechanisms above contribute to tumor innervation, tumors produce other substances that could possibly directly encourage axonogenesis. Although it has long been known that small extracellular vesicles (sEVs) generated by cancer cells aid in the formation of tumors, new research indicates that these sEVs may also have axonogenic properties [56]. Exosomes are vesicles with a rich cargo (DNA, RNA, proteins, and lipids) that range in size from 50 to 150 nm [57]. Neurite outgrowth is stimulated by the release of EphrinB1‐rich tumor exosomes in PC12 cells. This is significantly attenuated by compromised EphrinB1 expression or function. mEERL tumors that overexpress EphrinB1 exhibit a significantly higher degree of innervation compared with those exhibiting basal EphrinB1 expression [58]. Coculture experiments in vitro involving PC12 cells and sEVs derived from cervical cancer cell have yielded a notable observation: the presence of robust neurite outgrowth [59]. In accordance with the data presented, oncogene‐transformed fallopian tube cell lines and sEVs extracted from high‐grade serous carcinomas caused PC12 neurite outgrowth in vitro, with a comparable effect observed [23]. However, this response was not elicited by sEVs derived from normal fallopian tube cell lines [60]. sEVs may play a crucial part in the communication process with neural cells within the TME. Furthermore, it is plausible that newly transformed cancer cells may initiate this signaling process at an early stage of tumorigenesis.

### 4.2 The Landscape of Tumor Innervation: Density Dynamics and Morphological Heterogeneity

The interaction between nerves and tumors exhibits pronounced spatial and morphological heterogeneity. Analysis of nerve density across cancer types reveals considerable variability. Several solid malignancies, including prostate, pancreatic, gastric, and head and neck cancers, are frequently characterized by hyperinnervation, defined by an increased density of autonomic (sympathetic and parasympathetic) and sensory nerve fibers increases within and around the tumor tissue compared with the corresponding normal tissue (Table 1) [11, 12, 15, 45]. This phenomenon is often an active process driven by tumor‐derived axon guidance molecules and neurotrophic factors. Conversely, other cancers, such as basal cell carcinoma and colorectal cancer, may present with an overall alternation in density of specific kind of nerve or a functional denervation in specific compartments, suggesting a context‐dependent role for nerves [14]. These observations highlight that nerve density is a dynamic feature rather than a universal hallmark of cancer, and its functional impact depends on tumor type, nerve subtype, and anatomical location.

**TABLE 1:** Patterns of tumor innervation across cancer types.

Cancer type	Key markers used	Direction of change and key features	Key clinical associations	References
Prostate cancer	TH, VAChT, βIII‐tubulin	Sympathetic nerves (tumor margin), parasympathetic nerves (tumor core)	Poor prognosis, metastasis	[11]
Pancreatic	PGP9.5, TH, VAChT	Overall innervation, active axonogenesis	PNI, pain, poor survival	[13, 16]
Gastric	PGP9.5, TH	Intratumoral nerves	Poor prognosis, metastasis	[12, 61]
Head and neck	TH, TUJ1	Neuronal phenotype switching, neo‐innervation	PNI, Local recurrence	[45]
Breast cancer	PGPP9.5, NGF	Sensory and sympathetic fibers	Lymph node metastasis, poor survival	[15]
Non‐small cell lung cancer	PGP9.5, TH, CGRP	Sensory and sympathetic innervation	Metastasis, poor survival	[62]
Colorectal cancer	βIII‐tubulin, VAChT	Autonomic innervation with heterogeneous distribution	Invasion, immunosuppression	[63, 64]

Abbreviations: PGP9.5, protein gene product 9.5 (pan‐neuronal); PNI, perineural invasion; TH, tyrosine hydroxylase (sympathetic); VAChT, vesicular acetylcholine transporter (parasympathetic); βIII‐tubulin (TUJ1), pan‐neuronal.

In addition to overall changes in nerve density, the spatial distribution of nerves within tumors exhibits profound intratumor heterogeneity (ITH). In carcinomas such as those of the prostate and stomach, nerve fibers are often enriched at the invasive margin and in the peritumoral stroma, with lower density in hypoxic and necrotic tumor regions [11, 12]. This spatial organization suggests that nerves may facilitate local invasion and serve as conduits for cancer cell dissemination, contributing to PNI. Furthermore, the origin of tumor‐associated nerves is heterogeneous. While most nerves originate from local tissue innervation, evidence from prostate cancer models indicates that NPCs from the CNS can contribute to the sympathetic nerve supply in the TME [47].

Morphologically, tumor‐associated nerves are not merely passive bystanders but undergo active remodeling. This includes axonogenesis, the sprouting of new nerve fibers, and neural reprogramming, as demonstrated by the transition of sensory nerves to adrenergic neo‐neurons in head and neck cancer [45]. These newly formed fibers often exhibit an immature phenotype, characterized by increased expression of growth‐associated protein‐43 (GAP‐43) and dependence on specific neurotrophic factors for survival. In the CNS, gliomas develop a unique and distinct morphological structure known as tumor microtubes (TMs) [65]. These are ultra‐long, actin‐based membrane protrusions that are functionally and morphologically distinct from neuronal axons, yet they form a network that connects glioma cells over long distances, facilitating invasion, intercellular communication, and treatment resistance [66, 67]. The presence of TMs highlights that tumors can develop their own neural‐like network infrastructure, separate from endogenous nervous system of the host.

The assessment of tumor‐associated nerves relies heavily on immunohistochemistry combined with quantitative image analysis. Pan‐neuronal markers, including Protein Gene Product 9.5 (PGP9.5) and βIII‐tubulin (TUBB3), label axons of all types. Specific neuronal subtypes can be distinguished using antibodies against tyrosine hydroxylase (TH) for sympathetic fibers, vesicular acetylcholine transporter (VAChT) or choline acetyltransferase for parasympathetic fibers, and calcitonin gene‐related peptide (CGRP) or substance P for sensory fibers [68]. Nerve quantification is typically performed using two approaches: the hot‐spot method, which entails counting nerve fibers in predefined areas of highest density, or whole‐slide digital analysis, which provides a comprehensive overview of nerve distribution across the entire tumor section [69]. The hot‐spot method is often favored for its clinical relevance and association with aggressive features, whereas whole‐slide analysis minimizes sampling bias and more effectively captures ITH. Several challenges complicate the accurate quantification of tumor innervation. These include the difficulty of distinguishing delicate unmyelinated nerve endings from other stromal structures, the phenotypic plasticity of nerves in the TME, which may result in aberrant or mixed neurotransmitter expression and complicate the accurate classification of nerve subtypes, and the challenge of determining whether observed increases in nerve density are causal or consequential in the context of tumor progression.

### 4.3 Nerve–Tumor Interaction

**FIGURE 3:**
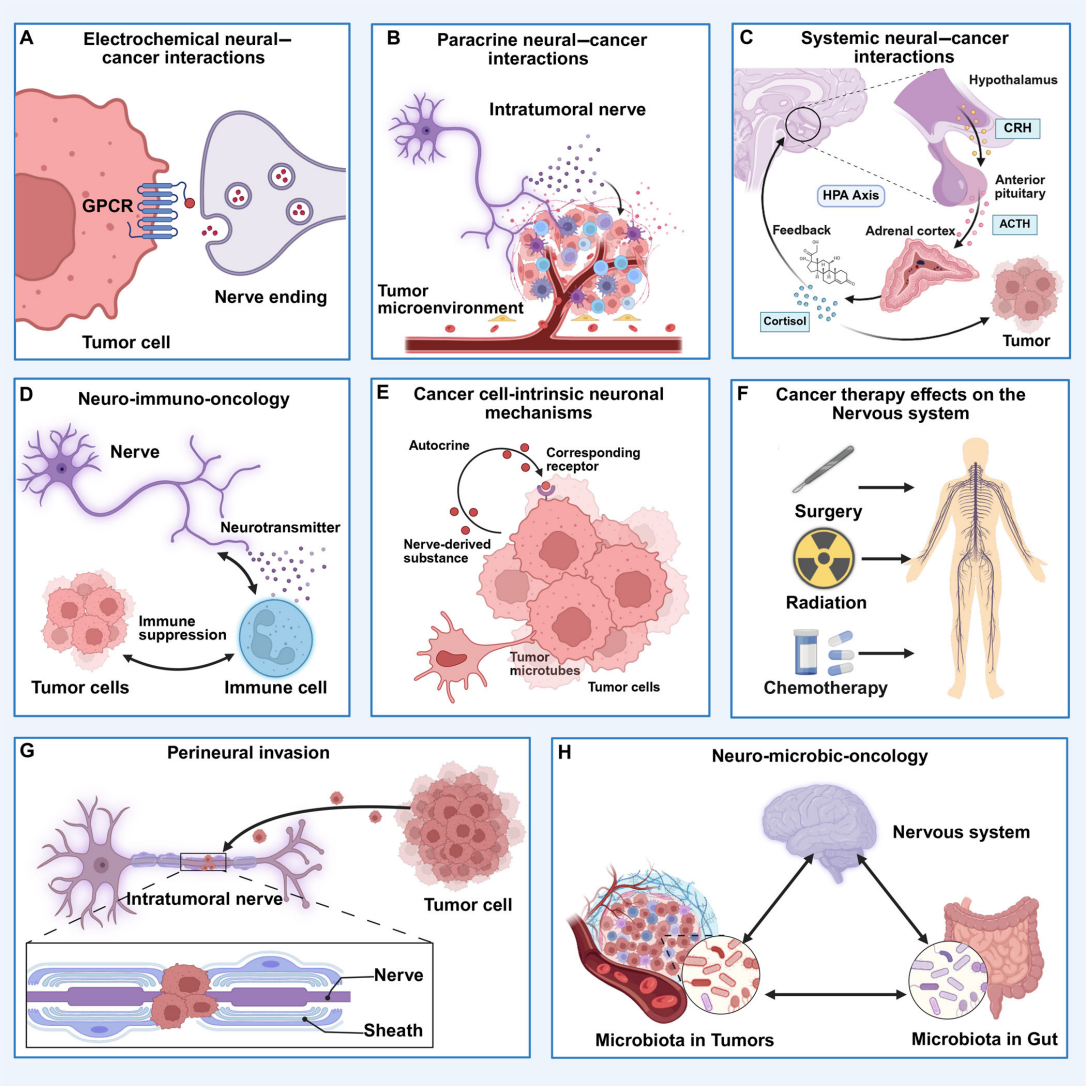
The nerve–tumor interaction. The nervous system and tumor could interact with each other in the following way. (A) Electrochemical interactions including neuron–cancer synapse. (B) Paracrine interactions from nerves to cancer cells, directly or through signaling with cells in the tumor microenvironment. (C) Nerve‐derived such as circulating neurotransmitters or neuropeptides that can influence cancer pathogenesis directly or indirectly such as through altered immune system function. Reciprocally, cancers can influence the nervous system at a distance through circulating factors or altered afferent neural signals. (D) Three‐way interactions between neurons or nerves, cancer cells, and immune cells can modulate anticancer immunity and procancer inflammation. (E) Cancer cells may leverage cell‐intrinsic signaling and other processes classically associated with neural cells. (F) Cancer therapies can profoundly alter nervous system function, including impaired function of various types of peripheral nerves and impaired cognitive function. (G) Tumors can invade nerves leading to PNI to facilitate cancer survival, immune escape, and metastasis. (H) Microbe may be an important component in nerve–tumor interaction. This figure was created using BioRender (https://biorender.com/). ACTH, adrenocorticotropic hormone; CRH, corticotropin‐releasing hormone; GPCR, G protein‐coupled receptors; HPA axis, hypothalamic–pituitary–adrenal axis.

As the specific mechanisms by which the nervous system influence cancer cells vary across tissues, summarizing the modes of nerve–tumor interaction may provide a unified framework for understanding cancer neuroscience. Nerve–tumor interactions are bidirectional and complicated. Monje et al. classified the interactions between the nervous system and cancer into four categories: electrochemical neural–cancer interactions, paracrine neural–cancer interactions, systemic neural–cancer interactions, and cancer therapy effects on the nervous system [2]. Then in 2023, Winkler et al. expanded these four categories to six, incorporating neuro‐immuno‐oncology and cancer cell‐intrinsic neuronal mechanisms [22]. In this review, we further categorize the nerve–tumor interaction into two additional modes: PNI and neuro‐microbic‐oncology (Figure 3).

#### 4.3.1 The Nerve–Tumor Interactions Proposed by Monje and Winkler

The intricate interplay between the nervous system and cancer manifests through several distinct yet interconnected mechanisms, beginning with direct electrochemical neural–cancer interactions where primary brain tumors and brain metastases from cancers like breast cancer and melanoma integrate into neural circuits by forming bona fide synapses with neurons (Figure 3A). These neuron‐to‐glioma synapses, mediated primarily by calcium‐permeable α‐amino‐3‐hydroxy‐5‐methyl‐4‐isoxazolepropionic acid (AMPA) receptors, allow for activity‐dependent depolarization of tumor cells and the secretion of growth‐promoting factors like neuroligin‐3 (NLGN3), thereby directly stimulating oncogenic pathways and promoting brain invasion [70, 71]. This synaptic integration is not static, as activity‐regulated signals such as NLGN3 and BDNF can promote the further establishment and maintenance of these malignant synapses over time, with synaptic gene expression increasing in recurrent glioblastoma [70, 72, 73]. Beyond direct synapses, paracrine neural–cancer interactions represent a widespread mechanism where secreted factors and neurochemicals profoundly influence tumor progression (Figure 3B). These include neurotrophic factors like NGF, BDNF, and glial cell line‐derived neurotrophic factor (GDNF), as well as neurotransmitters such as acetylcholine, glutamate, and norepinephrine, which collectively promote tumor growth through their respective receptors on cancer cells (Table 2) [74]. In CNS cancers, factors like NLGN3 [75, 76], BDNF [72, 77], and glucose‐regulated protein 78 (GRP78) [78] have been specifically implicated in promoting tumor initiation, growth, and proliferation. BDNF–TrkB signaling modulates the intensity of postsynaptic currents, while neuronal activity‐dependent paracrine signaling of NLGN3 increases the expression of postsynaptic receptor genes in glioma. This interaction establishes a feed‐forward loop that further enhances malignant progression [76, 79]. In addition to direct paracrine interactions between tumor cells and neural cells, they may also interact via other components within the TME. Norepinephrine released from adrenergic nerves activates β2‐ARs, thereby inducing cancer‐associated fibroblasts to produce elevated levels of NGF. NGF, in turn, promotes further intratumoral nerve sprouting, collectively driving the progression of colorectal cancer [80]. In breast cancer, tumor‐associated neurons form tunnel nanotubes (TNTs) that directly deliver functional mitochondria to cancer cells. This transfer depends on signaling through the epidermal epidermal growth factor receptor (EGFR) and its ligands, leading to actin cytoskeleton remodeling and TNTs formation. The acquisition of neuronal mitochondria significantly enhanced the oxidative phosphorylation (OXPHOS) capacity of the cancer cells, promoted a stem‐like state, and bolstered resistance to oxidative stress, thereby facilitating metastasis [81].

**TABLE 2:** Key neurotransmitters, receptors, and signaling pathways in cancer neuroscience.

Signaling molecule/pathway	Receptors	Downstream pathways	Effect on tumor/TME	References
Norepinephrine	β1/2‐AR (GPCR)	PKA, EPAC, Src	Promotes proliferation, angiogenesis, metastasis, immunosuppression	[82, 83]
Acetylcholine	mAChR (M1,M3) (GPCR)	MAPK/ERK, PKC	Promotes proliferation, survival	[84, 85]
Glutamate	NMDA, AMPA (Ionotropic)	Ca2+ influx, CREB	Promotes synaptic integration, brain metastasis, proliferation	[70, 71, 86]
GABA	GABA‐A (Ionotropic), GABA‐B (GPCR)	Cl− influx, cAMP	Mostly inhibitory on neurons; complex immunomodulatory	[87, 88]
CGRP	CLR/RAMP1 (GPCR)	cAMP, PKA	Promotes angiogenesis, pain, immunomodulation	[89, 90]
NGF	TrkA, p75NTR	PI3K/Akt, MAPK, NF‐κB	Axonogenesis, cancer cell survival, pain, PNI	[44, 61]
BDNF	TrkB	PI3K/Akt, MAPK, PLCγ	Neuronal plasticity, cancer cell survival, metastasis	[72, 91]

Abbreviations: AKT, protein kinase B; AMPA, α‐amino‐3‐hydroxy‐5‐methyl‐4‐isoxazolepropionic acid receptor; AR: adrenergic receptor; BDNF, brain‐derived neurotrophic factor; cAMP, cyclic adenosine monophosphate; CGRP, calcitonin gene‐related peptide; CLR, calcitonin receptor‐like receptor; CREB, cAMP response element‐binding protein; EPAC, exchange protein directly activated by cAMP; ERK, extracellular signal‐regulated kinase; GABA, gamma‐aminobutyric acid; GPCR, G protein‐coupled receptor; mAChR, muscarinic acetylcholine receptor; MAPK, mitogen‐activated protein kinase; NF‐κB, nuclear factor kappa‐light‐chain‐enhancer of activated B cells; NGF, nerve growth factor; NMDA, N‐methyl‐d‐aspartate receptor; p75NTR, p75 neurotrophin receptor, RAMP1, receptor activity‐modifying protein 1, Src, Sarcoma family kinase, TME, tumor microenvironment; PI3K, phosphatidylinositol 3‐kinase; PKA, protein kinase A; PKC, protein kinase C; PLCγ, phospholipase C gamma; PNI, perineural invasion; TrkA, tropomyosin receptor kinase A; TrkB, tropomyosin receptor kinase B.

At the level of the organism, systemic neural–cancer interactions are primarily orchestrated by the hypothalamus and its major outputs, namely, the HPA axis and the autonomic nervous system (ANS) (Figure 3C). These interactions regulate systemic physiological processes relevant to cancer. In this context, circulating mediators such as corticotropin‐releasing factor (CRF) and glucocorticoids can exert diverse, and sometimes opposing, effects on different tumors. For instance, CRF can transiently inhibit apoptosis and enhance motility in breast cancer, while suppressing proliferation in gliomas. Furthermore, CRF can also remodel the TME by influencing angiogenesis and immune cell function. Dysregulated HPA axis activity and glucocorticoid secretion are common in cancer patients and are associated to increased mortality and the promotion of metastatic colonization [92, 93, 94]. The ANS, as a major neural output, directly impacts cancer and stromal cells through adrenergic and cholinergic signaling, affecting processes from endothelial cell metabolism and angiogenesis in prostate cancer to metastasis in breast cancer [82, 95]. The concept of neuro‐immuno‐oncology is based on the presence and activity of nerves within the TME, which modulate tumor‐associated immune responses in a critical manner (Figure 3D). For instance, norepinephrine signaling can mediate immunosuppression by promoting T lymphocyte exhaustion and regulating myeloid‐derived suppressor cells and macrophage recruitment. In addition, neurotransmitters such as gamma‐aminobutyric acid (GABA) and CGRP can bind to receptors on immune cells to decrease antitumor immunity. Furthermore, blockade of β‐ARs has been shown to not only inhibit tumor progression but also enhance responses to immune checkpoint inhibitors, including anticytotoxic T‐lymphocyte‐associated protein 4 (anti‐CTLA‐4) therapy. These findings establish neuromodulation as a promising strategy for combinatorial cancer immunotherapy [96, 97, 98, 99, 100, 101].

Furthermore, cancer cells themselves have been observed to exhibit cancer cell‐intrinsic neuronal mechanisms, forming multicellular, functional, and resistant networks through ultra‐long, neurite‐like membrane protrusions known as TMs (Figure 3E). These structures facilitate intercellular communication via gap junctions and the exchange of molecules. In both pediatric and adult high‐grade gliomas, subpopulations of these interconnected tumor cells can exhibit autonomous, oscillatory calcium transients, acting as “hub” cells that generate depolarizing currents to drive tumor growth. This demonstrates that cancer cells can hijack neurodevelopmental programs to increase their capacity to proliferate and disseminate [66, 67, 70, 71, 102]. Finally, it is crucial to acknowledge the effects of cancer therapies on the nervous system. Chemotherapy, targeted therapies, and radiotherapy can lead to significant neural toxicity, including cognitive dysfunction in the CNS and sensory, motor, and autonomic neuropathies in the PNS (Figure 3F). This is due to mechanisms such as the dysfunction of neural precursor and stem cell populations, dysregulation of hippocampal neurogenesis, disruption of myelin homeostasis and plasticity, and direct damage to synaptic connectivity. This highlights the reciprocal and often detrimental impact of anticancer treatments on neural homeostasis and function [103, 104, 105, 106, 107, 108, 109].

While the frameworks established by Monje and Winkler comprehensively delineate the interactions between established tumors and the nervous system, they do not fully encompass two critical, clinically significant processes that represent distinct modes of nerve–tumor crosstalk. To provide a more holistic understanding, we now introduce and elaborate two additional dimensions of interaction: PNI, an active process of tumor dissemination along nerves, and the neuro‐microbic‐oncology axis, which incorporates the influence of the microbiota into the brain–tumor communication network.

#### 4.3.2 Perineural Invasion

PNI represents a distinct and critical mode of nerve–tumor interaction, defined not merely by anatomical proximity but by an active process in which tumor cells invade into the perineurium, the protective sheath surrounding nerves. Modern definitions have evolved from the classic “tumor in, around, and through nerves” to more precise criteria, such as tumor cells involving at least 33% of the nerve circumference or being present within any of the three layers of the nerve sheath (epineurium, perineurium, endoneurium) [110]. This histopathologic finding is a recognized independent prognostic factor in multiple staging systems, correlating strongly with local recurrence and poor survival (Figure 3G).

The initiation of PNI is driven by specific alterations in the TME. Hypoxia, a hallmark of solid tumors, upregulates hypoxia‐inducible factor‐1α, which in turn promotes the expression of key PNI mediators like C–X–C motif chemokine ligand 12 (CXCL12) and its receptor C–X–C motif chemokine receptor 4 (CXCR4) [111]. Hyperglycemia has been shown to promote the expression of NGF and its receptor TrkA, creating a neurotrophic gradient [112]. Additionally, inflammatory signals, such as those from pancreatitis‐associated proteins, can activate the JAK/signal transducer and activator of transcription (STAT) pathway, further facilitating PNI in pancreatic cancer [113].

The molecular execution of PNI is a coordinated, multistep process driven by several principal axes. The neurotrophic axis, primarily mediated by tumor and stromal cell‐derived factors such as NGF and GDNF, promotes cancer cell survival and directs migration by binding to their cognate receptors TrkA and Ret, respectively, on the cancer cell surface [44, 103]. Simultaneously, the chemotactic axis, involving ligand–receptor pairs such as CXCL12/CXCR4 and CX3CL1/CX3CR1, establishes a potent chemical gradient that guides the tumor cells along the nerve track [111]. Finally, to facilitate physical invasion through the protective nerve sheath, the matrix remodeling axis is activated, wherein tumor cells and associated fibroblasts release matrix metalloproteinases (MMPs) to degrade the ECM and the perineurial barrier [114]. These three axes operate in concert to enable the active, directional spread of cancer cells along nerves.

A major clinical consequence of PNI is neuropathic cancer pain. The intimate contact between cancer cells and the nerve sheath leads to the local release of algogens and the upregulation of pain‐related ion channels, such as transient receptor potential ankyrin 1 on sensory neurons, resulting in the release of substance P and CGRP, which drive peripheral sensitization and nociceptive signaling [115, 116]. Recognizing PNI as a distinct mode of interaction reframes it from a passive pathological finding to an active, orchestrated metastatic process, justifying the development of specific therapies targeting the tumor–nerve adhesion and invasion mechanisms.

#### 4.3.3 Neuro‐Microbic‐Oncology

The complex communication and regulation system between the gut and brain, called the gut–brain axis, comprising a bidirectional signaling network that involves neurons, hormones, immune cells, and the microbiota [117, 118]. As a consequence, the gastrointestinal (GI) tract influences on nervous system function, and vice versa. Through the ANS and the HPA axis, the brain controls gut function. Norepinephrine is released under stressful situations, which promotes the growth of intestinal pathogens [119]. Additionally, the microbiota releases metabolites and products, neuroactive substances, and hormones that travel through the vagus nerve, circulatory system, immune system, and ENS to affect the brain [120]. The vagus nerve, as the longest nerve of the ANS, can be considered as the core of gut–brain axis. Additionally, the induction, training, control, and function of both innate and adaptive immune response as well as local immunity are intimately linked to the microbiome [121]. Within the GI tract, cytokine secretion increases intestinal permeability, thereby facilitating the systemic dissemination of inflammatory molecules and their subsequent effects on the CNS [122]. Thus, the role of gut–brain axis in immune and homeostasis regulation is important.

There is growing evidence that tumor patients have an imbalance in the gut–brain axis, primarily through changes in immune system activity, host cell death and proliferation, and host metabolism [123] (Figure 3H). Porphyromonas gingivalis has been associated with an increased risk of pancreatic cancer [124]. P. gingivalis inhibit apoptosis through binding to Toll‐like receptor receptors, and activate NF‐κB and JAK/STAT3 pathway [125]. Additionally, P. gingivalis produces oncometabolites including oxygen radicals and acetaldehydes, which can lead to mutagenesis, DNA damage, and chronic inflammation, collectively contributing to cancer development [126]. Additionally, there are notable variations in β‐diversity, the firmicutes‐to‐bacteroides ratio, and the relative abundance of Verrcomicrobia and Akkermansia when fecal samples from glioma patients and animal models are compared with those from healthy controls [127]. Therefore, it is possible to speculate that microbiota could be leveraged to enhance the efficacy of current anticancer therapies, given the growing evidence that particular microbial taxa improve the efficiency of various treatment modalities against malignancies. The microbiota may be used as biomarkers to forecast the prognosis and responsiveness of patients [128]. Nevertheless, further research is necessary to ascertain the precise role of gut–brain axis components in tumor treatment [129]. Conceptualizing this axis as a separate category moves the field toward microbiome‐modulating interventions, such as probiotics, dietary strategies, fecal microbiota transplantation, to improve cancer outcomes, a strategy not encompassed by traditional neuro‐pharmacological approaches.

## 5 Cancer Neuroscience: Remodeling the Tumor Ecosystem

The diverse modes of nerve–tumor interaction described above do not operate as isolated phenomena, rather, they converge to reshape the tumor and its surrounding microenvironment into a dynamic system. Through these interactions, neural elements actively reprogram tumor progression, cellular fate, metabolic states, and intercellular communication networks. This remodeling extends beyond local innervation density to encompass spatial organization, functional plasticity, and crossorgan integration. In this context, the tumor is no longer viewed as a mass merely infiltrated by nerves, but as an ecosystem in which neural circuits, stromal components, immune populations, and cancer cells coevolve. Importantly, the manifestation of this remodeling differs between the CNS and PNS, reflecting the distinct physiological niches.

### 5.1 CNS Cancer Neuroscience

The aforementioned interaction mechanisms do not function in isolation. Instead, they synergistically reshape the TME through distinct pathways within both CNS and PNS, collectively establishing an ecosystem that supports tumor progression. During remodeling of the TME, tumors disrupt the signals such as cytokines [130], metabolites [131], hormones [132], hypoxia [18], tissue composition, and tissue stiffness [133]. Researching of the tumor‐related physiological signals integrating with the brain may help us to understand the dynamical change of the neural representations of cancer and the progress of cancer initiation, growth, progression, and metastasis from a extended timescale. Although there is no direct evidence regarding the temporal sequence of CNS activity and tumor development, tumor can cause diverse changes in circulating hormone levels before becoming clinical palpable [134]. These observations suggest that alterations in the CNS may represent an early event in cancer progression.

**FIGURE 4:**
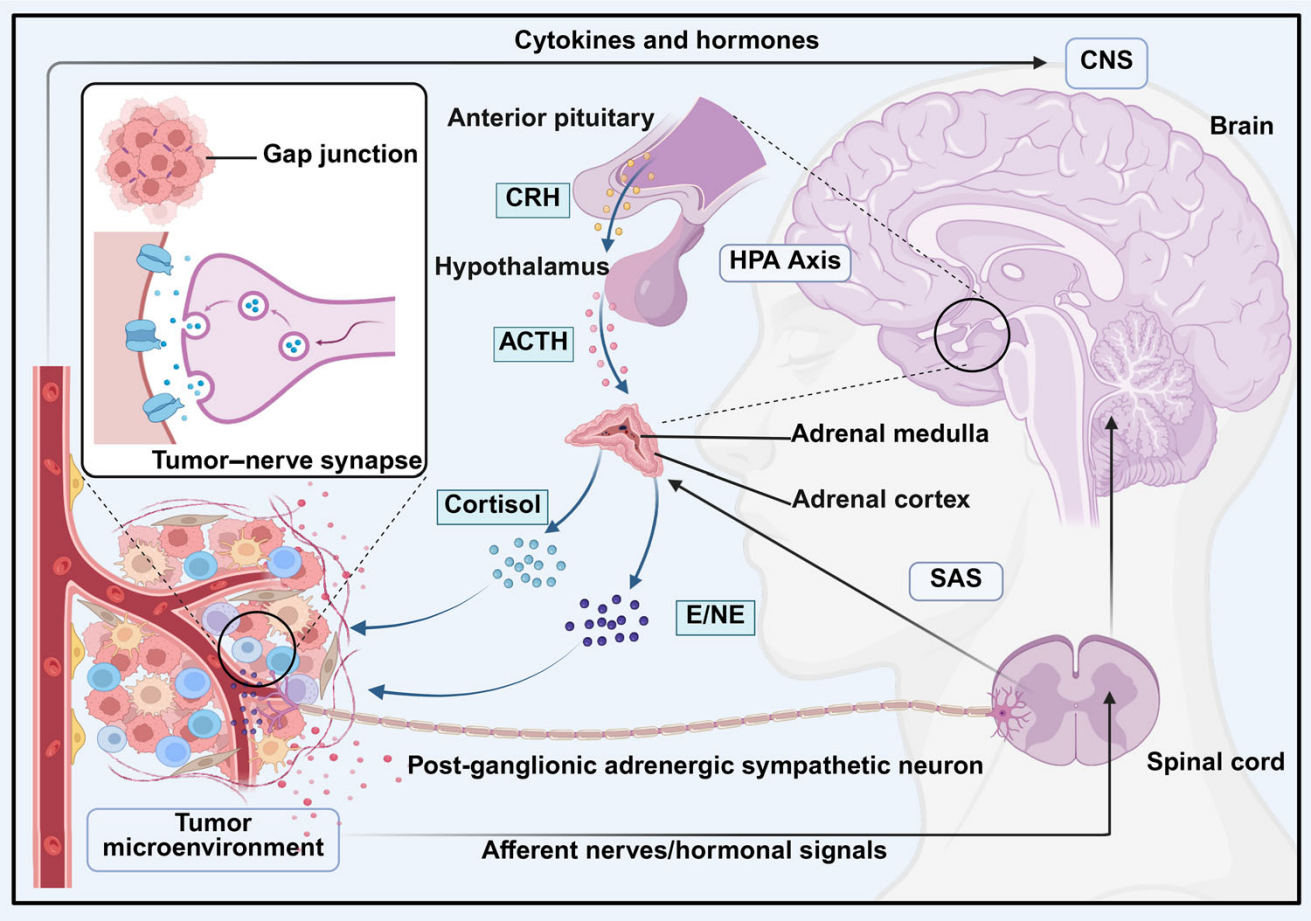
CNS cancer neuroscience. Specific brain areas indirectly connect with solid tumors and support their development. Efferent neural inputs from the brain can travel through central neuroendocrine systems such as the hypothalamic–pituitary–adrenal (HPA) axis or the sympatho–adrenal system (SAS). Tumors generate their own autonomic nerve network through the outgrowth of pre‐existing adrenergic or cholinergic nerve fibers in a process known as tumor axonogenesis to regulate cancer cells and other components of the tumor microenvironment. This figure was created using BioRender (https://biorender.com/). ACTH, adrenocorticotropic hormone, E, epinephrine; CNS, central nervous system; CRH, corticotropin‐releasing hormone; HPA, hypothalamic–pituitary–adrenal axis; NE, norepinephrine; SAS, sympatho–adrenal system.

Because of the unique niche of the brain microenvironment, only certain tumor cells, particularly metastatic cells originating outside the brain, can survive in this environment [3]. Thus, tumor cells in the brain hijack normal neural and neurodevelopmental mechanisms to adapt, progress, and develop multicellular networks [66, 70, 71, 135, 136] (Figure 4). This procedure shows that brain tumors uses preexisting relationships to establish and sustain itself rather than developing new operational principles [137]. Nerve–tumor synapses can promote glioma growth and invasion, particularly in aggressive and poor‐prognosis subtypes such as diffuse midline glioma and glioblastoma, as well as in certain non‐neural cancers [70, 71]. Besides, several neurotransmitter receptors are expressed on neuroblastoma cell membranes [138]. In addition to direct synapse formation, paracrine signaling, such as insulin‐like growth factor (IGF1), GRP78, BDNF, Semaphorin 4F (Sema4F), and NLGN3, have been implicated in brain tumor progression and invasion [76, 139, 140]. The metalloprotease a disintegrin and metalloproteinase 10 (ADAM10) regulates the neuronal activity‐driven shedding of NLGN3. In animal models, it has been demonstrated that blocking ADAM10 expression inhibits the formation of gliomas [75, 91]. Furthermore, recent evidence suggests that neuronal release of IGF1 can promote the development of IDH‐wildtype gliomas in mice [140]. Seizures and neuronal hyperexcitability are common in glioma patients [141], a phenomenon resulting from multiple factors, including neuronal hyperexcitability, edema, and tumor infiltration, which disrupt neurovascular coupling and neuronal network integrity [142]. The neurotransmitter glutamate, which is secreted by glioma cells, can promote glioma invasion and growth via an autocrine mechanism [86, 143, 144] and induce epilepsy via both AMPA and N‐methyl‐d‐aspartate (NMDA) receptors on neurons, highlighting the promising role of antiepileptic drugs and AMPAR inhibitors in glioma treatment [144]. TMs, existing in glioma, communicate via intercellular Ca waves depending on gap junction connections [67] (Figure 4). This network maintains intercellular homeostasis and contributes to resistance to cytotoxic and other therapies [65, 67, 145, 146] and plays a critical role in tumor progression [102].

The complex relationships between the tumor and the CNS exemplify the significant challenges that remain to be addressed. Our understanding of brain neuroscience is expected to advance through research on tumor vascular biology, neural–tumor coregulation of the BBB, dysregulated ion channels in tumor cells, and other neural processes that govern brain tumor biology.

### 5.2 PNS Cancer Neuroscience

**FIGURE 5:**
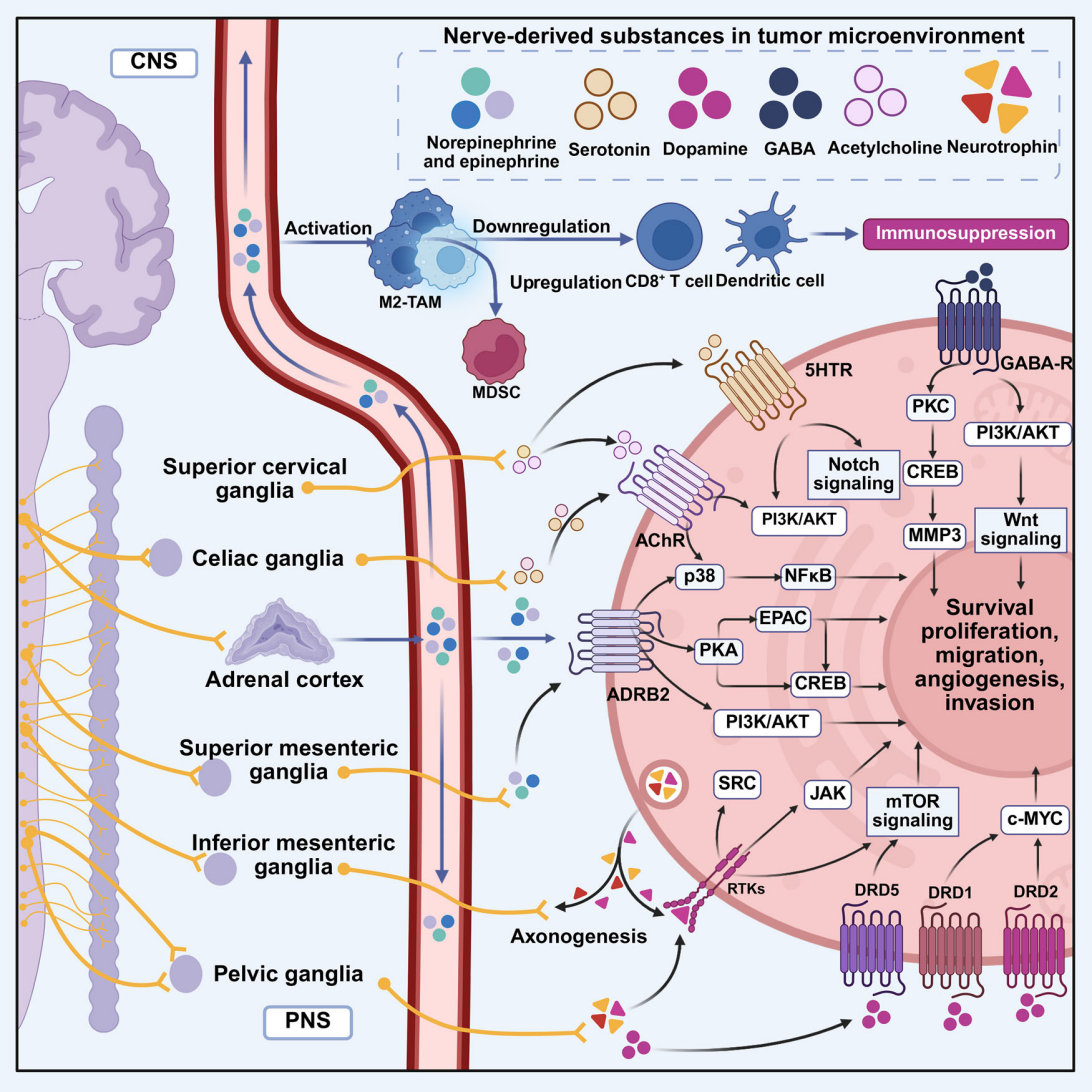
PNS cancer neuroscience. The peripheral nervous system significantly contributes to tumor microenvironment regulation through the sympathetic nervous system (SNS), parasympathetic nervous system (PNS), and enteric nervous system (ENS). During development, the superior cervical ganglia (SCG), the prevertebral sympathetic ganglia, the celiac ganglia (CG), the superior mesenteric ganglia (SMG), the inferior mesenteric ganglia (IMG), and the pelvic ganglia (PG) extend axonal projections to supply and interact with various peripheral organs and tissues. These neurons could release various kinds of neurotransmitters such as norepinephrine and dopamine into TME to regulate cancer development. Neurogenesis within the SNS and PNS is stimulated by neurotrophins released by tumor, leading to axonogenesis and the formation of neural networks that influence cancer development and spread. This figure was created using BioRender (https://biorender.com/). CG, celiac ganglia; CNS, central nervous system; IMG, inferior mesenteric ganglia; PG, pelvic ganglia; PNS, parasympathetic nervous system; SCG, superior cervical ganglia; SMG, superior mesenteric ganglia.

In the periphery, different types of nerves innervate different tumors. As mentioned in Section 4.1, the nerves within the TME may reflect the organ's inherent innervation pattern, arise from stem or progenitor cell differentiation, or be induced by tumor cells to develop within the TME. Numerous studies across diverse cancer types provide compelling evidence that the nervous system plays a critical role in the initiation of neoplastic diseases outside the CNS [22] (Figure 5). In non‐small cell lung cancer, infiltrating nerves and neural elements within the TME have been shown to modulate tumor metabolic reprogramming, promote proliferation and invasion through neurotrophic signaling, and influence responses to immunotherapy by altering tumor immune landscapes [147]. In breast cancer, tumor‐associated innervation drives prometastatic programs and is linked to enhanced metastatic potential via sensory and adrenergic circuits that facilitate neural recruitment and neuropeptide‐mediated stimulation of metastatic gene expression [148]. In colorectal cancer, PNI is a prominent feature of disease progression and is associated with poor prognosis, reflecting a critical form of nerve–tumor crosstalk whereby cancer cells co‐opt enteric and autonomic neural pathways to support invasion and dissemination [63]. Collectively, these findings indicate that neural regulation of tumor behavior is not restricted to rare neurogenic neoplasms but is a pervasive mechanism influencing the progression, metastasis, and clinical outcomes of major solid tumors [62].

In the TME, ARs, which are GPCRs, are abundantly expressed [149]. Activation of ARs promotes protumorigenic signaling in tumor cells, whereas antagonists inhibit cell survival [150], proliferation [151], invasion [152], and evasion of apoptosis [153] (Figure 5). Adrenergic signaling also impacts other components of the TME, such as immune cells and endothelial cells, inducing angiogenesis [82] and metastasis [15]. Cholinergic neurons contribute to neo‐angiogenesis, migration, proliferation, and cell survival in head and neck squamous cell carcinoma [154], lung [155], gastric [156], brain [157], and prostate [158] cancers. Nevertheless, even within specific tissues, it is essential to precisely delineate the contributions of diverse neurotransmitters derived from parasympathetic and sympathetic activity (Figure 5). In pancreatic cancer, cholinergic signaling has been reported to inhibit tumor growth, whereas adrenergic signaling promotes it [16]. GABA, a major inhibitory neurotransmitter in the CNS, extends its influence beyond the CNS and significantly modulates peripheral metastasis, tumor growth, and antitumor immunity [87, 88, 159]. Tumor or immune‐derived signals sensitize injury receptor neurons, which exhibit heightened neural excitability and release various neuropeptides that promote tumor cell proliferation [46, 160, 161], metastasis [46], and lead to immune escape [160, 161, 162, 163, 164]. Besides, tumor‐associated neurons in breast cancer models can directly transfer functional mitochondria to cancer cells via TNTs [81]. This neuron‐to‐tumor cell mitochondrial transfer significantly enhances the OXPHOS capacity of cancer cells, properties of stem cells, and resistance to oxidative stress during metastasis. This finding provides a direct metabolic explanation for the neural addiction of cancer and establishes neurons as a key, functionally active metabolic niche within the TME.

In conclusion, research on the diverse ways in which neurons and malignant cancer cells interact within their microenvironment across various tissues and organs remains ongoing. However, the connections between peripheral tumors and brain networks, as well as their potential responses to electrochemical neurotransmission, are still largely unexplored. Compelling evidence indicates that distinct neuronal subtypes contribute to the development of malignant tumors in multiple cancers, including those of the oral cavity, head and neck, GI tract, colon, rectum, prostate, breast, and pancreas. Investigating the effects of membrane depolarization and activity‐mediated neurotransmission on these cancer cells represents a promising area of future research. The influence of the nervous system on tumor progression extends well beyond the local lesion, and these systemic interactions underlie numerous debilitating complications in cancer patients, profoundly affecting both quality of life and clinical outcomes.

## 6 Cancer Neuroscience: Cancer‐Associated Comorbidity

The neural remodeling of the tumor ecosystem described above extends far beyond local tumor progression and microenvironmental restructuring. As the nervous system become chronically reprogrammed in the context of malignancy, its systemic regulatory functions are progressively destabilized. Cancer therefore induces not only structural and functional alterations within the tumor niche, but also widespread perturbations across central and peripheral neural networks that govern mood, cognition, metabolism, cardiovascular homeostasis, and pain perception. These cancer‐associated comorbidities should not be viewed merely as secondary complications of tumor burden or treatment toxicity. Conceptualizing these clinical manifestations within the framework of cancer neuroscience provides a mechanistic bridge between tumor biology and patient experience and underscores the necessity of integrating neurobiological regulation into comprehensive oncologic care.

### 6.1 Anxiety and Depression

Major depressive disorder and anxiety are very common comorbidities in cancer patients. Recent studies indicate that depression affects approximately 24% of cancer patients and is more prevalent in advanced stages of the disease, as measured by the Patient Health Questionnaire‐9 [165]. Several tumor types are associated with anxiety or depression, with lung tumors often causing these symptoms via endocrine paraneoplastic syndromes, and pancreatic tumors inducing severe cytokine‐mediated depression [166].

The expression of indoleamine 2,3‐dioxygenase‐1 (IDO1), an enzyme involved in the kynurenine pathway that degrades serotonin (5‐HT) and generates neuroactive metabolites in the brain, is strongly implicated in the prodromal symptoms of depression in cancer patients [167]. The IDO1 inhibitor epacadostat has been shown to significantly improve mood compared with vehicle controls. These findings suggest that IDO1 inhibition may represent a therapeutic strategy for alleviating depressive symptoms in patients with pancreatic cancer [167]. Nevertheless, it remains essential to provide comprehensive psychosocial and supportive care to both patients and their families.

### 6.2 Anorexia and Cachexia

Cancer‐related anorexia/cachexia (CAC) is a complex syndrome characterized by progressive weight loss, tissue wasting, and reduced food intake, accompanied by declines in adipose tissue and skeletal muscle mass, as well as poor performance status [168]. And the incidence of CAC varies by tumor type: gastric (85%), pancreatic (83%), non‐small cell lung (61%), small cell lung (57%), prostate (57%), and colon (54%) [169, 170, 171]. CAC is therefore recognized as a multifaceted adaptation involving a spectrum of behavioral and physiological changes and is associated with poor clinical outcomes and reduced quality of life [172].

Functional impairment of the hypothalamic processes that typically regulate eating behavior is closely associated with cancer‐related anorexia [173]. Leptin, an adipokine, acting on a specific hypothalamic receptor, promotes satiety via downstream neuropeptides such as neuropeptide Y [172, 174, 175]. Cytokines, including interleukin (IL)‐1, have been shown to induce anorexia by enhancing leptin expression and release, thereby mimicking the hypothalamic effects of leptin [175, 176, 177]. IL‐1, as one of the most potent anorectic cytokines, exerts its strongest effects when administered into the ventromedial hypothalamus [178]. The substance under investigation can exert its effects through two primary mechanisms. First, it may directly cross the BBB. Second, it can stimulate the ascending fibers of the vagus nerve, leading to CNS production of IL‐1 [179]. Additionally, IL‐1 can induce anorexia by promoting CRF synthesis in the hypothalamus, an effect that can be partially blocked by CRF antibodies [180]. TNF‐α, produced by monocytes, macrophages, and tumor cells, is capable of crossing the BBB and stimulating the ascending fibers of the vagus nerve, thereby contributing to anorexia via peripheral or central mechanisms [181, 182, 183]. Lactate, a metabolic byproduct of tumor cells, activates glucose‐responsive neurons in the ventromedial hypothalamus, thereby reducing satiety [184, 185, 186, 187]. Furthermore, cancer treatments that induce nausea and vomiting can contribute to anorexia [188].

### 6.3 Cognitive Dysfunction

A decrease in cognitive function, including learning, attention, executive functions, memory, multitasking, and processing speed, is the hallmark of cognitive dysfunction, related to cancer therapy, commonly referred to as “chemobrain” or “chemofog” [189], and is associated with chemotherapy, targeted therapies, immunotherapy, and hormone therapy [189, 190]. Among breast cancer patients, the prevalence of self‐reported cognitive difficulties related to chemotherapy ranges from 21 to 34% [191]. These findings indicate that a substantial proportion of patients continue to experience persistent cognitive symptoms [192].

It is recognized that several factors can increase the permeability of the BBB during chemotherapy, potentially leading to neurotoxicity. Systemically administered chemotherapeutic agents, such as Adriamycin, have been shown to induce oxidative stress, which disrupts the structure of the BBB [193]. Anthracyclines and platinum‐based drugs are among the anticancer agents capable of generating cellular reactive oxygen species (ROS) in nontumor tissues [194]. In animal models, intravenous administration of cyclophosphamide and doxorubicin has been associated with oxidative damage to RNA and DNA in the hippocampus [195]. Breast cancer patients treated with anthracyclines exhibit elevated levels of tumor necrosis factor (TNF) receptors, which correlate with declines in cognitive function [196]. Furthermore, even at low doses, cisplatin has been shown to reduce dendritic spine density in the hippocampus and induce neuronal apoptosis, thereby impairing neurogenesis in rodents [197].

### 6.4 Chemotherapy‐Induced Peripheral Neuropathy

Chemotherapy‐induced peripheral neuropathy (CIPN) is a common and treatment‐induced clinical problem that affect the sensory, motor, and autonomic nerves, resulting in pain, dysesthesia, and paresthesia, predominantly in the limbs [198]. Peripheral neuropathy frequently arises as an adverse effect of various cancer therapies, including vinca alkaloids, taxanes, platinum compounds, and proteasome inhibitors [104]. Approximately 30–40% of patients receiving neurotoxic chemotherapy develop CIPN, with onset and severity correlating with dosage—particularly the cumulative dose and frequency of administration—as well as additional risk factors such as smoking and diabetes [104, 199].

Although the underlying mechanisms remain unclear, previous basic research has focused on neurotoxic damage to dorsal root ganglion sensory neurons, involving microtubule dysfunction, oxidative stress, inflammation, DNA damage, direct nerve injury, and altered ion channel signaling [200]. Voltage‐gated sodium channels, particularly Nav1.7 and Nav1.8, which mediate the initiation, amplification, and conduction of action potentials in nociceptive neurons, can be upregulated and hyperactivated by chemotherapy. This hyperactivity can lead to spontaneous currents, resulting in increased pain sensitivity and neuropathic pain [201]. Chemotherapy also enhances the expression of chemokines, including C–C motif chemokine ligand 2 (CCL2) and CX3CL1, in sensory neurons [202]. Proinflammatory mediators, including IL‐6, adenosine triphosphate (ATP), and chemokines, are also produced by Schwann cells following nerve injury, maintaining neuroinflammation through the recruitment of macrophages [203, 204, 205]. Furthermore, paclitaxel induces opening of the mitochondrial permeability transition pore in axons, resulting in loss of mitochondrial membrane potential, increased ROS production, decreased ATP levels, calcium release, and mitochondrial swelling [206]. Chemotherapeutic agents have also been shown to impair the antioxidant defense systems of sensory neurons, leading to elevated ROS generation, damage to cellular components, lipid peroxidation, mitochondrial membrane depolarization, and reduced ATP synthesis [207]. In areas of the skin that correspond to the most severe symptoms of CIPN, the combined impact of the previously discussed processes seems to result in a reduction of intraepidermal nerve fibers and Meissner's corpuscles [208]. These structural alterations can compromise neuronal survival and function, contributing to the development of CIPN. CIPN represents a dose‐limiting toxicity that highlights the vulnerability of peripheral nerves to chemotherapeutic agents.

### 6.5 Cancer Pain

Pain is a common comorbidity that may prompt the initial diagnosis of cancer and is a significant source of fear for patients throughout the disease course [209]. The experience of pain, coupled with inadequate analgesia, can profoundly impair emotional well‐being and functional status. This may lead to increased depression, anger, anxiety, and cognitive dysfunction, ultimately diminishing patients’ quality of life [210]. Notably, cancer‐related pain often persists at severe levels during treatment and can remain substantial for at least 3 months following curative therapy [211].

In bone and pancreatic cancers, pain is recognized as a significant clinical complaint. The most frequent cause of cancer‐related pain is bone metastasis [212, 213]. Within this microenvironment, acidosis driven by disseminated tumor cells disrupts normal bone remodeling by mimicking the functions of both osteoblasts and osteoclasts [214, 215, 216]. Activation of acid‐sensing ion channels and TRP channels in this acidic microenvironment may contribute to peripheral sensitization by disturbing skeletal homeostasis [217, 218]. Furthermore, IL‐1β, released by tumor and stromal cells, can enhance prostaglandin production by upregulating cyclooxygenase‐2 expression in macrophages, thereby increasing their sensitization [219, 220]. At sites of active bone remodeling, nerve density is elevated, and increased intraosseous pressure within the bone microenvironment activates mechanoreceptors and mechanotransducing osteocytes, which in turn sensitize primary afferents [221, 222, 223]. Macrophage infiltration into peripheral sensory ganglia has also been frequently observed in models of nerve injury. Research in this field has highlighted the importance of this neuro‐immune interface in the development of chronic inflammatory demyelinating polyneuropathy [224].

Pain is the third most common complaint in pancreatic cancer patients. Because pancreatic cancer cells and associated inflammatory cells release NGF, PNI may represent a key feature of the disease. When these cancer cells invade the perineurium of nearby intrapancreatic nerves, pancreatic neuropathy and visceral pain can occur [225, 226, 227]. In the context of OCSCC, a malignancy frequently associated with pain, the superior gingival fluid (SGF) from human OCSCC cells contains proteases capable of activating and sensitizing parathyroid hormone‐related peptide‐expressing sensory neurons. Intraperitoneal injection of SGF in murine models induces profound and sustained mechanical allodynia [228]. Thus, targeting these neurotrophic pathways offers the dual benefit of alleviating pain while potentially impeding tumor progression, highlighting the therapeutic potential of modulating nerve–tumor interaction.

### 6.6 Other Systemic Comorbidities: Sleep Disturbance, Fatigue, Metabolic Syndrome, and Cardio‐oncology

Other prevalent and interconnected systemic comorbidities, including sleep disturbances, fatigue, metabolic syndrome, and cardiovascular complications, profoundly impact patient quality of life and survival. These conditions frequently share underlying neurobiological and systemic pathophysiological mechanisms, often mediated by dysregulation of the HPA axis, the ANS, and chronic inflammation.

Sleep disturbance in cancer patients, characterized by insomnia, nocturnal awakenings, and disrupted circadian rhythms, is closely linked to somatic symptoms such as pain and psychological distress [229]. Critically, the dysregulation of diurnal cortisol rhythms, a marker of HPA axis dysfunction, is associated with both sleep maintenance problems and reduced long‐term survival, suggesting a shared neuroendocrine pathophysiology [230]. This disruption is further aggravated by cancer therapies, which can directly alter sleep–wake cycles through mechanisms involving inflammatory activation and psychological stress [231]. Cancer‐related fatigue, one of the most prevalent and debilitating treatment‐related symptoms, exhibits a complex etiology that intersects with these same pathways [232]. It is mechanistically associated with dysregulated proinflammatory cytokines, such as IL‐1, IL‐6, TNF‐α, which can activate the HPA axis and alter 5‐HT metabolism, ultimately reducing somatomotor drive and perpetuating a state of fatigue [233]. Furthermore, a shift in autonomic balance toward sympathetic dominance, reflected by elevated norepinephrine levels and reduced heart rate variability, is a recognized contributor to the persistence of fatigue [234, 235].

The dysregulation of neuroendocrine and inflammatory systems also underlies the relationship between cancer and metabolic syndrome [236]. The dysfunction of the HPA axis and chronic low‐grade inflammation in metabolic syndrome collectively create a systemic environment favorable to tumors: aberrant glucocorticoid secretion patterns are not only associated with poor patient prognosis but can also directly promote tumor metastasis, while the persistent inflammatory state further enhances the release of tumor‐promoting neuropeptides by inducing neuroinflammation and sensitizing nociceptive sensory neurons [46, 94, 237]. Therefore, metabolic syndrome can be viewed as a critical accelerator that, by converging multiple dysregulations in hormonal, neural, and immune systems, provides an ideal pathophysiological niche for the neural addiction of cancer.

The field of cardio‐oncology addresses the critical challenge of cardiovascular disease resulting from cancer therapies, a comorbidity that significantly impacts overall prognosis [238]. Cardiovascular autonomic dysfunction (AD) is a key manifestation, often arising from the neurotoxic effects of chemotherapeutic agents such as platinum compounds, taxanes, and vinca alkaloids [239]. These agents can induce oxidative stress, mitochondrial damage, and direct injury to small autonomic fibers, leading to parasympathetic denervation and sympathetic predominance [240]. Radiotherapy targeting thoracic or cervical regions can cause fibrosis and inflammation of neural structures, including the vagus nerve, further disrupting baroreflex function and heart rate control [241, 242]. The proinflammatory state induced by both chemotherapy and radiotherapy, characterized by the release of cytokines such as TNF‐α and IL‐6, exacerbates this autonomic imbalance by modulating central and peripheral neural circuits [243]. Management strategies increasingly include cardioprotective pharmacotherapy, such as beta‐blockers and angiotensin‐converting enzyme inhibitors (ACEIs), which have been shown to mitigate anthracycline‐induced cardiotoxicity and improve outcomes in cancer patients [244].

In summary, sleep disturbance, fatigue, metabolic syndrome, and cardiovascular complications constitute a constellation of interrelated comorbidities in cancer patients. Their pathogenesis is deeply rooted in the systemic dysregulation of neural, endocrine, and immune circuits, highlighting the need for integrated, multidisciplinary approaches to patient care that target these shared underlying mechanisms.

## 7 Cancer Neuroscience: Targeting Nerve–Tumor Interaction

A comprehensive understanding of neuro‐tumor interactions and their clinical consequences ultimately underscores a clear objective: the development of novel therapeutic strategies that target these pathways to both directly suppress tumors and simultaneously alleviate patient suffering. Due to the close correlation between nerves and tumor development, tumor therapies targeting nerves have emerged as a focus of clinical research. As early as the 19th century, denervation was reported to inhibit tumor growth. With the advancement of medical technology and deeper understanding of the composition and function of nerves within tumors, surgical denervation has been gradually refined, but the role of denervation surgery in clinical practice has been investigated in only a limited number of tumor types, such as some tumors of the GI tract. Notably, the recurrence rate in patients who underwent simultaneous gastrectomy and vagotomy was lower than in those who underwent gastrectomy alone, supporting the concept that denervation may serve as an adjuvant to conventional therapies to benefit patients [12]. In addition to surgical interventions, pharmacological inhibition of neural signaling represents an effective strategy for targeting nerves in tumor therapy. As mentioned previously, beta‐adrenergic antagonists target adrenergic signaling and can be applied not only in cardiovascular disease but also in the treatment of various tumors, including pancreatic cancer, breast cancer, and melanoma [245] (Table 1). Clinical studies investigating tricyclic antidepressants and selective 5‐HT reuptake inhibitors for glioblastoma treatment are also ongoing [246]. Although clinical trials of many new neurochemical modulators are underway, and repurposing existing drugs with known safety profiles may accelerate the integration of neurotherapy with existing clinical therapies, the specificity of achieving effective drug concentrations around tumors without affecting physiological neurological function is a major challenge in the new use of old drugs.

### 7.1 Targeting Local Nerve–Cancer Interaction

As described before, cancer neuroscience as an emerging but rapidly evolving field that enhances our understanding of cancer as a systemic disease and has the potential to revolutionize cancer therapy. It is clear that an integrated framework spanning preclinical and clinical‐translational research is required in order to achieve progress. In addition to expertise in neurology and oncology, the development and adoption of novel technologies will be essential. Using methodological frameworks informed by cancer neuroscience, it will be crucial to examine pharmacological and nonpharmacological therapies throughout the course of the disease, with thorough clinical characterization in both animal models and clinical trials.

Strategically, because the mechanisms underlying nerve–tumor interactions largely mirror physiological states of the nervous system, the primary considerations in drug development are specificity and effective concentration, alongside careful assessment of potential neurological side effects in patients.

#### 7.1.1 Targeting Electrochemical Neural–Cancer Interactions

As outlined above, neuronal electrical activity is essential for optimal neurodevelopment. Accumulating evidence indicates that such electrical input plays a critical role in promoting cancer proliferation, particularly in cases of brain tumors. These findings provide a compelling rationale for the consideration of antiepileptics as a therapeutic modality. Although these agents are conventionally used to prevent seizures, they may also confer additional anticancer effects by reducing neuronal activity. Preliminary work has investigated use of the antiepileptics perampanel [71], talampanel [247], valproic acid [248], and levetiracetam [248] in cancer treatment, although further investigation is required (Table 3).

**TABLE 3:** Drugs of targeting nerve–tumor interaction.

Targets	Drug	Tumor type	Function	State	References
RAMP1	Rimegepant	Oral squamous cell carcinoma; acute myeloid leukemia	Proliferation	Pre‐clinical	[90, 249, 250]
GABA receptor	Baclofen	Breast cancer; hepatocellular carcinoma	Immune therapy sensitization	Phase II (NCT00493428); Phase III (NCT00516503)	[251]
Cholinergic receptor	Pirenzepine	prostate cancer; glioblastoma	Proliferation; metastasis; chemotherapy‐induced peripheral neuropathy	Phase II (NCT05488873)	[154, 252, 253, 254]
	Atropine	Colorectal cancer; breast cancer	Proliferation; migration; epithelial–mesenchymal transition	Preclinical	[255, 256, 257]
β2‐AR	Atenolol	Gastric cancer; breast cancer; colorectal cancer; hepatocellular	Proliferation; metastasis	Phase IV (NCT05106179)	[258, 259, 260]
	Bisoprolol	Breast cancer	Cardiotoxicity; cardio‐oncology; cachexia	Phase III (NCT04588935)	[261, 262]
	Carvedilol	Glioblastoma; breast cancer; prostate cancer	Growth; metastasis; cardiotoxicity	Early Phase I; Phase II (NCT03861598; NCT02944201)	[263, 264, 265]
	Propranolol	Breast cancer; non‐small cell lung cancer; bladder cancer	Neoadjuvant	Phase II (NCT02596867);	[266, 267, 268, 269, 270]
NK1R	Aprepitant	Gallbladder; lung cancer; prostate cancer; glioblastoma; ovarian cancer	Proliferation, migration, invasion; nausea	Phase II (NCT00212602, NCT01046461); Phase III (NCT01536691, NCT00572572); Phase IV (NCT02532634, NCT06007586);	[271, 272, 273, 274, 275]
Dopamine receptor	Benztropine	Breast cancer; colorectal cancer; gastric cancer	Epithelial–mesenchymal transition; proliferation; metastasis; CIPN	Preclinical	[276, 277, 278, 279, 280, 281]
	Thioridazine	Lung cancer; ovary cancer; colorectal cancer; breast cancer; head and neck squamous cell carcinoma	Drug resistance; autophagy; apoptosis	Phase I (NCT02096289)	[282, 283, 284, 285, 286, 287]
	ONC201	Glioma, pancreatic cancer; breast cancer	Proliferation; metastasis; drug resistance; apoptosis; DNA damage; acute myeloid leukemia	Phase I (NCT02250781,); Phase II (NCT03034200, NCT03791398, NCT06012929); Phase III (NCT05580562)	[288, 289, 290, 291, 292, 293, 294]
	Trifluoperazine	Colorectal cancer; lung cancer; osteosarcoma	Ferroptosis; proliferation; apoptosis; metastasis; drug resistance	Preclinical	[295, 296, 297, 298, 299, 300]
NMDAR	Memantine	Hepatocellular sarcoma; breast cancer; lung cancer	Metastasis; cognitive dysfunction; CIPN	Early Phase I (NCT04217694); Phase II (NCT04033419, NCT04801342, NCT03194906, NCT01260467); Phase III (NCT04737967)	[301, 302, 303, 304, 305, 306]
5HTR	Ondansetron	Breast cancer; head and neck squamous cell carcinoma; lung cancer	Nausea; vomit	Phase II (NCT04508400, NCT01031498); Phase III (NCT01536691, NCT03606369, NCT03578081, NCT06208917, NCT05872893)	[307, 308, 309, 310, 311, 312]
TRPV1	Capsaicin	Colorectal cancer; gastric cancer; cervical cancer; breast cancer	Metastasis; CIPN; proliferation; EMT; invasion	Phase II (NCT03317613, NCT02037464); Phase III (NCT03794388, NCT04704453, NCT00003610)	[313, 314, 315, 316, 317, 318, 319, 320]
VIPR	ANT008	Pancreatic ductal adenocarcinoma	Immune therapy sensitization	Preclinical	[321]
	ANT308	Leukemia	T cell activation	Preclinical	

Abbreviations: 5HTR, 5‐hydroxytryptamine receptors; NK1R, neurokinin receptor 1; NMDAR, N‐methyl‐d‐aspartate receptor; RAMP1, receptor activity modifying protein 1; TRPV1, transient receptor potential cation channel subfamily V member 1; VIPR, vasoactive intestinal polypeptide receptor 1; β2‐AR, beta‐2 adrenergic receptor.

A significant challenge in the management of tumor‐related epilepsy involves interactions between pharmaceutical agents, particularly those employed in cancer therapy, which can complicate treatment regimens. Interactions may occur at any stage of a drug's pharmacokinetic profile, from absorption to elimination. The most clinically significant drug–drug interactions are generally related to hepatic metabolism and excretion. Enzyme‐inducing drugs have been shown to accelerate the elimination of coadministered agents, whereas enzyme‐inhibiting drugs can reduce their metabolism. For example, it has been demonstrated that phenytoin, carbamazepine, oxcarbazepine, and phenobarbital function as enzyme inducers. Consequently, the metabolism and clearance of numerous chemotherapeutic agents, including corticosteroids, paclitaxel, cyclophosphamide, etoposide, topotecan, nitrosoureas, doxorubicin, and methotrexate, can be increased by this property [142].

Recent studies have shown that, in contrast to conventional postsurgical anti‐inflammatory therapy with dexamethasone, genetic targeting of GAP‐43 effectively reduces the formation and activity of TMs, thereby mitigating surgery‐induced tumor growth. Following a single cycle of temozolomide chemotherapy, significant associations were identified between the heterogeneity of TMs formation and interconnection, both intra‐ and intertumoral, and the response to therapy. The integration of tumor cells within TMs networks was identified as a strong predictor of chemotherapeutic resistance [65].

#### 7.1.2 Targeting Paracrine Neural–Cancer Interactions

**FIGURE 6:**
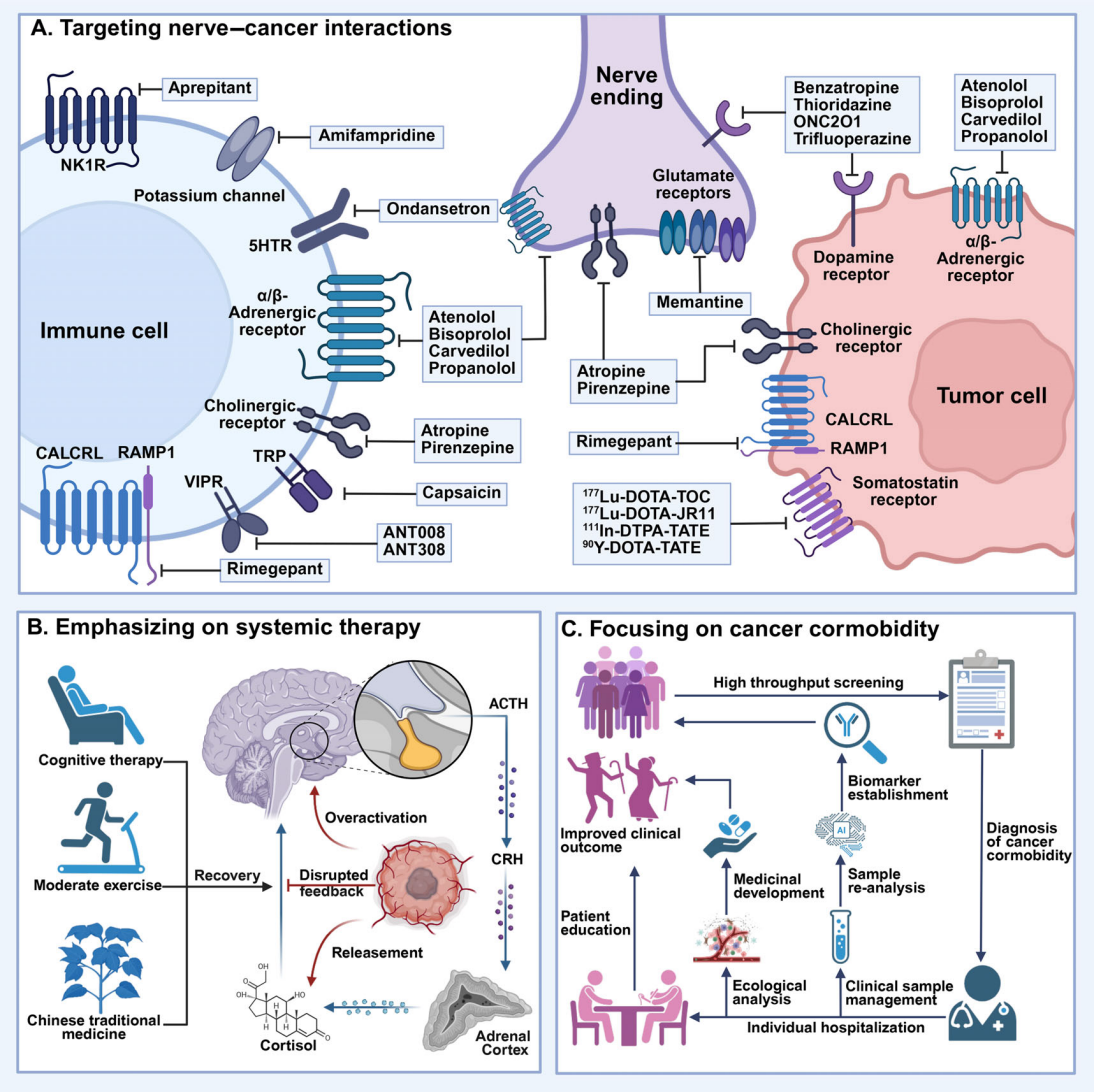
Therapies targeting cancer neuroscience. (1) Neurons release neurotransmitters that affect immune cell activity and promote cancer cell growth, while cytokines released by immune cells enhance neuronal activity and stimulate cancer cell aggressiveness. However, blocking the related receptor via corresponding antagonists may benefit cancer therapy. These receptors could also be implicated in cancer targeted therapy; (2) with the progress in conception, systemic cancer therapy may be a potent strategy. Cognitive therapy, moderate exercise, and Chinese traditional medicine may relief cancer‐associated stress and recover the imbalanced HPA axis; (3) possible therapeutic templates for tumor comorbidity. This figure was created using BioRender (https://biorender.com/). β2‐AR, beta‐2 adrenergic receptor; 5HTR, 5‐hydroxytryptamine receptors; NK1R, neurokinin receptor 1; NMDAR, N‐methyl‐d‐aspartate receptor; RAMP1, receptor activity modifying protein 1; TRPV1, transient receptor potential cation channel subfamily V member 1; VIPR, vasoactive intestinal polypeptide receptor 1.

The tumor‐promoting potential of these neurochemicals highlights their value as therapeutic targets. The repurposing of existing neuroregulatory medications as anticancer therapies is under investigation (Figure 6). In both melanoma patients and preclinical models, treatment with the β1‐ and β2‐AR antagonist propranolol has been shown to inhibit tumor progression, suggesting that melanoma development may be driven by β1‐AR activation [322]. The combination of epinephrine and propranolol has been shown to reduce the in vitro proliferative activity of cancer cells (Table 3). This suggests that the AR signaling pathway may play a regulatory role in the antiproliferative effects of the catecholamine epinephrine [323]. MicroRNAs, including miR‐13p, miR‐103, miR‐107, miR‐147, and miR‐191 (in retinoblastoma), act as tumor suppressors by downregulating BDNF and/or its downstream signaling pathways [324]. Due to their excellent BBB permeability, neurochemical modulators may be particularly effective in treating brain tumors; however, their lack of tumor specificity and potential adverse effects pose significant challenges for clinical application.

Nerves have been shown to influence the TME and early cancer cells, thereby regulating growth and promoting metastasis. In this process, nerves act both as physical conduits and as mediators of vascular and lymphatic dissemination [325]. The stimulation of the parasympathetic nervous system has been demonstrated to increase tumor metastasis and invasion, thereby enhancing late‐stage cancer progression. In the context of gastric cancer cells, the activation of β‐adrenergic signaling has been observed to upregulate the expression of vascular endothelial growth factor and MMPs. This phenomenon has been shown to result in the upregulation of the STAT3 and the extracellular signal‐regulated kinase 1/2 (ERK1/2) and JNK–MAPK signaling pathways, which have been implicated in promoting metastasis in vivo [258]. TrkB has been demonstrated to induce epithelial–mesenchymal transition (EMT), and knockdown of Twist effectively blocks TrkB‐induced EMT, anoikis resistance, and tumor growth [326]. The E‐cadherin‐interacting protein ankyrin‐G binds to the neurotrophin receptor‐interacting MAGE homolog (NRAGE) and mediates anoikis regulatory signals. NRAGE has been shown to repress the p14ARF gene, thereby promoting anoikis resistance [327].

The transplantation of human breast cancer cells into immunodeficient mice, combined with treatment using either anti‐NGF antibodies or small interfering RNA targeting NGF, resulted in the inhibition of tumor growth and metastasis. This study evaluated the effects of these treatments on NGF‐stimulated breast cancer cells. The results demonstrated that these treatments led to a decrease in cell proliferation, increased apoptosis, and inhibition of tumor angiogenesis [328]. Consequently, monoclonal antibodies targeting NGF, such as fasinumab and tanezumab, are currently under development and have been clinically tested in nononcological populations [329]. Importantly, determining the optimal timing of administration and potential combination with other therapies is critical, as therapeutic strategies may aim either to prevent metastatic spread or to treat established metastatic tumors.

#### 7.1.3 Targeting Neuro‐Immuno‐Oncology

Since effective immune responses can eliminate malignant cells or impair their phenotypes and functions, immunotherapy, which enhances natural defenses to treat cancer, has been recognized as a viable strategy [330]. As previously noted, immune activity can be modulated by the nervous system. Therefore, combining neuroactive agents with cytotoxic therapies, immune checkpoint inhibitors, or cancer vaccines may represent a promising approach for cancer treatment (Figure 6).

Using a syngeneic mouse fibrosarcoma model, beta‐2 AR (ADRB2) signaling may change the composition of the TME in soft tissue sarcoma [331]. Propranolol treatment reduces tumor angiogenesis and enhances the antitumor response by increasing the infiltration of T cells into tumors. It has also been shown to potentiate the efficacy of anti‐CTLA4 therapy, highlighting its ability to improve the effectiveness of immune checkpoint inhibition (Table 3) [332]. The primary objective of the first Phase Ib/II clinical trial combining propranolol with pembrolizumab, an anti‐PD1 checkpoint inhibitor, was to determine the optimal dosage for a Phase II trial and evaluate the efficacy of this combination in patients with melanoma (NCT03384836). There is also evidence that β‐blockade can modulate the immune response to enhance the efficacy of cytotoxic therapies. In vitro, β‐AR signaling can directly increase tumor cell resistance to radiation when stimulated with the agonist isoproterenol [99].

We searched these clinical trials on ClinicalTrials.gov (https://clinicaltrials.gov/), using keywords including “cancer neuroscience,” “tumor innervation,” “perineural invasion,” “neuro‐immune oncology,” “β‐blocker cancer,” “neuron–glioma synapse,” “gut–brain axis cancer,” “neurotrophic factor tumor microenvironment,” and specific drug and receptor names.

### 7.2 Systemic Cancer Therapies

#### 7.2.1 Psychological Support and Exercise Intervention

Because tumor patients often suffer from depression and anxiety, psychological support and exercise interventions may be particularly beneficial, depending on the severity of symptoms. Nonpharmacological approaches such as music therapy, meditation, stress management, and yoga have been shown to improve outcomes in oncology patients [333]. Additionally, psychological interventions, including cognitive‐behavioral therapy, have been reported to improve quality of life, cortisol levels, self‐efficacy, sexual functioning, sleep quality, and reduce fatigue in cervical cancer patients [334]. Some guidelines recommend incorporating physical activity as part of the standard of care for pediatric cancer patients [335]. Overall, psychosocial interventions and structured physical activity represent effective nonpharmacological strategies for adolescents with cancer, helping to manage both acute and long‐term cancer‐related symptoms [336]. Cancer patients, particularly those with advanced disease, are more vulnerable than healthy individuals, highlighting the need for personalized exercise and psychosocial regimens [337]. Moreover, these interventions may be extended to family members, providing supportive care beyond the patient alone.

#### 7.2.2 Traditional Chinese Medicine

An important feature of traditional Chinese medicine (TCM) is its systemic therapeutic approach. This perspective aligns with the evolving understanding of tumors as complex systems rather than merely collections of disorganized cells. Several TCM interventions have been reported to alleviate stress, potentially restoring HPA axis function in a more regulated manner. For example, Lily (Lilium spp.) has demonstrated antidepressant and anxiolytic effects. Some studies have also suggested that these interventions may influence tumor progression, and experimental evidence has identified the active bioactive constituents responsible for these effects [338]. Saikosaponin D, a naturally occurring compound derived from herbal sources, has been demonstrated to to significantly reverse norepinephrine‐induced chemoresistance by downregulating ADRB2 expression [338]. 10‐Gingerol, a bioactive constituent isolated from the rhizome of Zingiber officinale, interacts with β2‐ARs, potentially disrupting adrenergic signaling and enhancing chemosensitivity. Baicalin, a major bioactive component of Scutellaria baicalensis Georgi, directly modulates β2‐AR activity, thereby inhibiting EMT in breast cancer [339]. The practice of TCM emphasizes the use of herbal remedies and natural products to provide a holistic therapeutic approach, offering a complementary perspective on cancer treatment.

### 7.3 Targeting Cancer‐Associated Comorbidity

In previous sections, we discussed the relationship between the nervous system and cardio‐oncology from a pathophysiological perspective, with less emphasis on specific clinical manifestations. In this section, we provide a concise overview of several common cardiovascular disorders and their corresponding management strategies (Figure 6).

Due to the high incidence of cancer‐associated hypertension, comprehensive consideration of preventive strategies, accurate diagnosis, and long‐term management is warranted. Compared with the diagnosis of hypertension in the general population, experts recommend out‐of‐office blood pressure (BP) measurements, such as ambulatory BP monitoring [340, 341]. However, these methods often require patients to wear the monitor for extended periods, which can be inconvenient. Management strategies include both pharmacological interventions and lifestyle modifications. ACEIs and angiotensin receptor blockers (ARBs) are regarded as first‐line agents in cancer patients [342]. For instance, in patients with renal cell carcinoma treated with sunitinib, the use of ACEIs or ARBs was associated with improved overall survival and effective BP control. Beta‐blockers also demonstrate efficacy in the management of resistant hypertension [244].

Heart failure (HF) is another common comorbidity in cancer patients, primarily arising as a consequence of cancer therapies. For example, anthracyclines are considered the most prevalent cause of HF among anticancer agents [343, 344], as they exert direct cytotoxic effects on cardiomyocytes via topoisomerase II‐mediated DNA damage, increased ROS production, and mitochondrial dysfunction [345, 346]. β‐Blockers have demonstrated cardioprotective effects by improving contractile function, metabolism, and myocyte survival. Carvedilol, nebivolol, alprenolol, and other β‐blockers can inhibit β1‐adrenergic signaling and transactivate β‐arrestin, thereby activating downstream receptor tyrosine kinase pathways and promoting prosurvival signaling in cardiomyocytes [347].

ACEIs also demonstrate favorable therapeutic effects. They are thought to downregulate angiotensin II levels, thereby upregulating the expression of neuregulin‐1, a ligand for EGFR, human EGFR 2 (HER‐2), and other receptor tyrosine kinases [348]. Furthermore, early administration of the anti‐IL‐6 monoclonal antibody tocilizumab has been shown to improve outcomes and enhance the safety profile without compromising anticancer efficacy [349].

Cancer therapies, including surgery, radiotherapy, systemic therapies, and chemotherapies, can act as stressors to tumor cells. The remarkable ability of tumor cells to adapt to these stressful environments is a major contributor to treatment resistance. Nerves can serve as a source of essential nutrients and modulate both tumor and stromal cell responses to microenvironmental stress. In gliomas, TMs—ultra‐long membranous protrusions composed of actin and microtubules—play a significant role in mediating treatment resistance. Given that myosin and microtubules constitute these microtubes, targeting their components may provide a therapeutic strategy. As microtubule inhibitors, taxanes and vinca alkaloids depolymerize microtubules, disrupt mitotic spindles at high doses, prevent proliferating cancer cells from completing mitosis, and result in chromosome condensation [350]. However, the limited BBB penetration of taxane‐based therapies represents a significant challenge for their use in CNS malignancies. For cancer types that thrive in nutrient‐scarce microenvironments, such as pancreatic cancer and high‐grade gliomas, and for the treatment of local recurrences, where tumor regrowth occurs in previously treated tissue with altered vascularization and nutrient supply, therapeutic strategies that target prosurvival mechanisms under microenvironmental stress may offer the greatest potential benefit.

Patients with cancer frequently describe experiencing excruciating pain at both the primary and metastatic locations. The existence of PNI has been associated with this occurrence, which is thought to be partially caused by the activities of neurotrophins and neuroregulatory proteins. The etiology of cancer pain is multifaceted, encompassing factors such as direct nerve invasion or compression by solid tumors. Additionally, neural toxicity, chemotherapy, and radiotherapy have been identified as contributing elements to the complex pathophysiology of cancer pain [351]. These neuronal signaling pathways are appealing therapeutic targets due to the significant morbidity of cancer pain and the well‐established fact that the upstream paracrine signaling proteins that modulate pain also have actions that promote tumor development. The goal of cancer pain management is to control the symptoms, but these therapeutic approaches may also stop the development of the tumor.

## 8 Cancer Neuroscience: On the Road

This review systematically traces the development and core advances in the emerging field of cancer neuroscience. We not only integrate existing frameworks of neuro–tumor interactions but also propose theoretical foundations for recognizing PNI and neuro‐microbic‐oncology as distinct interaction patterns. Extensive evidence indicates that the nervous system profoundly influences tumor initiation, progression, metastasis, and treatment resistance through diverse mechanisms, including electrochemical synapses, paracrine signaling, systemic neuroendocrine regulation, and neuro‐immune‐oncology. Whether through the hijacking of neural circuits by CNS tumors or the active innervation of peripheral tumors, these phenomena clearly demonstrate that the nervous system is a critical, indispensable component within the TME. This perspective elevates the understanding of tumor biology from a localized cellular pathology to a systemic disease affecting the entire organism.

Despite rapid advancements in this field, numerous critical questions remain unresolved. At the mechanistic level, the temporal roles of distinct neuronal subtypes in tumor evolution and their specific molecular coding remain poorly understood. The precise signaling networks governing neuro–immune crosstalk within tumors also require further elucidation. Overcoming technical bottlenecks is essential for advancing the field. Future studies should leverage spatial transcriptomics, single‐cell sequencing combined with optogenetics, and other cutting‐edge technologies to map the dynamic landscape of neuro–tumor interactions across both temporal and spatial dimensions. The development of novel organoid systems and in vivo models that more accurately recapitulate the coevolutionary processes of human neuro‐tumors will provide powerful platforms for mechanistic investigation.

Translating insights from cancer neuroscience into clinical practice represents both a major challenge and a unique opportunity. Current research priorities should emphasize precision in targeted therapies, including the exploration of synergistic combinations and optimal sequencing of neuromodulatory agents with chemotherapy, radiotherapy, immunotherapy, and other targeted treatments. Careful evaluation of safety concerns, particularly the risk of long‐term AD from neuromodulation, is essential. In the context of PNI and cancer pain, the development of highly specific and effective inhibitors of neurotrophic signaling pathways remains an urgent priority.

Looking ahead, advancing cancer neuroscience will require deep integration among neurobiologists, oncologists, immunologists, bioengineers, and clinicians. Establishing interdisciplinary collaboration platforms is essential to systematically decipher the heterogeneity of neural contributions across cancer types and to explore interventions targeting emerging pathways, such as the neuro‐microbiome axis. Ultimately, integrating neuro‐targeting into precision cancer therapy promises a transformative paradigm—shifting from merely suppressing tumor growth to comprehensively enhancing patient quality of life and survival outcomes.

## Author Contributions

W.L.J., T.W., and Z.K.D. were involved in the conception and design of the review. T.W. and Z.K.D. wrote the paper and prepared the figures and tables. W.L.J., Y.F.W., Z.Y.A., and S.Y.W. critically reviewed and edited the manuscript. All authors have read and approved the article.

## Funding

This work was financially supported by the High‐Level Talent Introduction Funds from the First Hospital of Lanzhou University.

## Ethics Statement

The authors have nothing to report.

## Conflicts of Interest

The authors declare no conflicts of interest.

## Data Availability

The authors have nothing to report.
